# How much does community-based targeting of the ultra-poor in the health sector cost? Novel evidence from Burkina Faso

**DOI:** 10.1186/s13561-018-0205-7

**Published:** 2018-09-04

**Authors:** Yvonne Beaugé, Jean-Louis Koulidiati, Valéry Ridde, Paul Jacob Robyn, Manuela De Allegri

**Affiliations:** 1 Institute of Public Health, Medical Faculty, Heidelberg University, Heidelberg, Germany; 2 IRD (French Institute for Research on Sustainable Development), CEPED (IRD-Université Paris Descartes), Universités Paris Sorbonne Cités, ERL INSERM, SAGESUD and University of Montreal, Public Health Research Institute (IRSPUM), Montréal, QC Canada; 3 The World Bank, Washington DC, USA

**Keywords:** Community-based targeting, Targeting, Ultra-poor, Pro-poor targeting, Activity-based costing, Burkina Faso, Costs, Economic evaluation

## Abstract

**Background:**

Targeting efforts aimed at increasing access to care for the poorest by reducing to a minimum or completely eliminating payments at point of use are increasingly being adopted across low and middle income countries, within the framework of Universal Health Coverage policies. No evidence, however, is available on the real cost of designing and implementing these efforts. Our study aimed to fill this gap in knowledge through the systematic assessment of both the financial and economic costs associated with designing and implementing a pro-poor community-based targeting intervention across eight districts in rural Burkina Faso.

**Methods:**

We conducted a partial retrospective economic evaluation (i.e. estimating costs, but not benefits) associated with the abovementioned targeting intervention. We adopted a health system perspective, including all costs incurred by the government and its development partners as well as costs incurred by the community when working as volunteers on behalf of government structures. To trace both financial and economic costs, we combined Activity-Based Costing with Resource Consumption Accounting. To this purpose, we consulted and extracted information from all relevant design/implementation documents and conducted additional key informant structured interviews to assess the resource consumption that was not valued in the documents.

**Results:**

For the entire community-based targeting intervention, we estimated a financial cost of USD 587,510 and an economic cost of USD 1,213,447. The difference was driven primarily by the value of the time contributed by the community. Communities carried the main economic burden. With a total of 102,609 ultra-poor identified, the financial cost and the economic cost per ultra-poor person were respectively USD 5,73 and USD 11,83.

**Conclusion:**

The study is first of its kind to accurately trace the financial and economic costs of a community-based targeting intervention aiming to identify the ultra-poor. The financial costs amounted to USD 5,73 and the economic costs to USD 11,83 per ultra-poor person identified. The financial costs of almost USD 6 represents 21% of the per capita government expenditure on health.

**Electronic supplementary material:**

The online version of this article (10.1186/s13561-018-0205-7) contains supplementary material, which is available to authorized users.

## Key messages

- This study fills an important knowledge gap on cost of community-based selection and targeting interventions
- Financial costs associated with community-based targeting in Burkina Faso amounted to USD 5,73 and the economic costs to USD 11,83 per ultra-poor person identified
- Economic costs amounted to USD 1,213,447 including the value of the effort contributed by volunteers, exceeding by two-folds the financial costs of USD 587,510

## Background

Universal Health Coverage (UHC) aspires to provide affordable and quality health care to all [1]. However, many low and middle income countries (LMICs) continue to rely on user fees which severely limits access to care and use of health services, especially for the poorest segments in a society [2]. In order to protect the most vulnerable people from financial hardship, some countries have opted for exempting the poorest from user fees [3] or for subsidizing health insurance premiums [4]. The identification and selection of the poor for user-fee exemption or insurance premium subsidization has been proven to be very challenging [5].

In the absence of universal criteria to define poverty and identify the poorest, countries have traditionally adopted various targeting methods. In the literature, the identification of beneficiaries is presented as the process of selecting the persons that benefit from a certain intervention. Targeting, on the other hand, is commonly used as a broader term which includes the identification as well as the actual act of allocating scarce resources to the poorest with the aim of achieving certain policy objectives to preserve or improve health equity [6].

In the health sector, most targeting experiences derive from those with the greatest health risks or the highest ability to benefit from the programs [7]. Those are generally the poorest [8] but can also be other groups of people such as the elderly, orphans or people with disabilities [9].

Generally, high-income countries use a form of means-testing and identify ultra-poor based on a certain income threshold. On the contrary, targeting strategies without a direct observation of income, such as proxy means testing (PMT) and community targeting, are predominantly used in LMICs [6]. While PMT uses a formal algorithm to identify households’ wealth, community based targeting (CBT) collects information from local leaders [10].

All targeting mechanism are prone to errors and generate costs [11]. Various studies have shown that there is no best solution to identify beneficiaries for targeting health benefits. Relative accuracy and cost effectiveness are said to be best achieved by a well-designed and implemented mechanism [9]. However, the scientific community continues to debate the relative costs and benefits of universal vs. targeted strategies in allocating resources to the poorest and alleviating poverty [12–14]. The question is whether the costs associated with the CBT program actually compensate for the savings accrued when offering subsidized or free health services only to a selected number of individuals.

These arguments are mostly postulated in the light of theoretical assumptions on the costs and benefits associated with targeting procedures, but are not substantiated by any extensive empirical evidence [15]. The literature suggests that CBT can potentially contribute to a reduction in administrative costs compared to other targeting methods [8, 16, 17], since the bulk of resources, such as time and expertise, are provided by community members who are usually not remunerated. Prior studies [18, 19] addressed the costs of pro-poor targeting programs briefly by focusing on the financial expenses and showed that engaging the poor to identify the poorest is not costly. However, both studies disregarded, amongst other components, the time volunteers (e.g. community members) invested into the targeting exercise. Prior studies which addressed the effectiveness of pro-poor targeting showed inconsistent results [20–23]. These evaluations have focused primarily on estimating how effective programs are in relation to how well all members of a certain target group are included, and members of the non-target group excluded from participating in a targeted program [20–22]. The available evidence, however, is almost completely silent as to whether targeting policies for the poor offer good value for money, as measured in terms the relationship between their costs and their benefits.

Towards this ends, our economic evaluation began to fill an important gap in knowledge by assessing both the financial and the economic costs associated with designing and implementing a pro-poor targeting project, with specific focus on the identification and targeting component integrated within the Performance Based Financing (PBF) intervention recently rolled out in Burkina Faso [24]. We plan to include these first cost estimates into more comprehensive cost-effectiveness analysis of pro-poor targeting policies in the future.

## Methods

### Study setting

This study took place in Burkina Faso, where a PBF intervention was launched in 2014 under coordination of the Ministry of Health (MoH) with financial support from the Health Results Innovation Trust Fund (HRITF), administered by the World Bank (WB). The Programme d’Appui Au Développement (PADS), Burkina Faso’s initiative to better coordinate and manage resources for the implementation of the National Health Development Plan, channeled the funding provided by the HRITF to the CBT intervention. The Society for Studies and Public Health Research (SERSAP), a for-profit consultancy firm, led the implementation of the CBT intervention. The objective of the intervention is to combine a supply-side (PBF) with a demand side (subsidy for the poorest) component to maximize health gains for the poorest. [25] In eight out of a total of 12 districts implementing PBF, healthcare providers receive payments based on a fixed unit price per service, plus a lump-sum to reimburse (at the expected average cost of treatment) for loss of income due to loss of user fees, by treating the ultra-poor for free. Thereby, it is ensured that health facilities are not disadvantaged by delivering care to the ultra-poor who do not pay the health providers directly [26].

The process of selecting and identifying the ultra-poor by community members, including the design and implementation of the structures, lasted from May 2014–January 2016. Across the eight districts, it covered 1,745,789 individuals, i.e. approximately 10% of the country’s total population (18.1 million) [27]. Additional file 1 provides details on the organization of the CBT selection process, under leadership by SERSAP. A total of 1172 Community Selection Committees (CSI) (gender-balanced) were set up across the eight districts at the village level to select the ultra-poor based on their profound knowledge of the population, and establish lists of the ultra-poor. The CBT selection processes were guided by the definition of an ultra -poor (indigent) person as “someone who is extremely socially and economically disadvantaged, unable to care for himself (herself) and who is without internal or external resources” [20] and was meant to result in the compilation of community-specific lists of the ultra-poor as individuals eligible to benefit from a user fee exemption.

The lists of the ultra-poor persons were verified by two entities, the Local Implementation Teams (ELMEO) and the Local Validation Groups (GVL). For this validation process, the respective teams used a list of 20 ultra-poor criteria which were initially developed in the context of the action research done in one district in Burkina (Ouargaye) [28]. The ELMEO randomly selected people from the list and verified whether these criteria were respected (external validation), before handing the list over to the GVL. The GVL then went through the entire list and checked whether each selected person fulfilled the ultra-poor criteria, then confirmed or rejected their ultra-poor status (internal validation). The WB developed a database of the ultra-poor in order to compile detailed information about them (e.g. village, full name, age, gender). Thirty-five enumerators collected these data, including digital pictures of the ultra-poor. The information was used to issue identity cards (produced in Vietnam) that had to be shown in order to receive basic primary, secondary and tertiary health care free of charge (fees and drugs) at all public health facilities within the implementation districts of the PBF [29].

Monitoring and Evaluation (M&E) was secured by the ELMEO, the Regional Directorate for Health and Social Action and the Regional Technical Assistants (ATR). Originally, it was envisioned that 15 to 20% of the population will be targeted and exempted from user fee payment. On average 6% of the total population were finally identified as the ultra-poor [29].

### Study design and conceptual approach

We conducted a retrospective partial economic evaluation [30] with the objective of estimating both the financial and the economic costs of the abovementioned community-based identification and targeting of the ultra-poor. We refer to our work as a partial economic evaluation, since we focused exclusively on documenting costs and not on establishing a relationship between the costs and consequences of the intervention. This decision is linked to two factors. First, we wanted to focus exclusively and carefully on the cost component to fill the important gap in knowledge on the real cost of targeting outlined earlier. Second, at the time of our study, the intervention was ongoing; hence, it was not yet possible to determine the extent to which targeting had actually contributed to an increase in health service utilization among the poorest and the related health service provision costs. Hence, we traced costs for the design phase (May 2014–August 2014) and the implementation phase (September 2014–January 2016), defining as implementation only the process of identifying and selecting the ultra-poor, not of providing healthcare services to them.

As financial costs (accounting costs), we defined all explicit financing transactions made by any of the concerned actors to carry out any activity related to either the design or the implementation of the intervention. These costs can be verified retrospectively on the accounting systems of the implementing agencies. As economic costs, we defined the real value of the resources consumed by the intervention.

We adopted a health system perspective, meaning that we aimed at tracing all costs incurred by the MoH and its partners, including development partners and implementing agencies. Costs incurred at the community level were included in the analysis insofar as the community was engaged as a volunteer agent to act on behalf of the Ministry as a formal implementing partner, effectively carrying out/substituting key intervention functions.

To trace both financial and economic costs, we combined Activity-Based Costing (ABC-approach) with Resource Consumption Accounting. ABC aggregates costs by activity, attributing indirect and support expenses to the individual activities [31]. Resource Consumption Accounting first itemizes (identification) and measures (measurement) the resources necessary to carry out a given activity and then values (valuation) the consumption of each resource for service provision and calculating or estimating the costs of each resource [32].

### Data collection

We carried out data collection over a period of six months from October 2016 to March 2017. We computed all costs in US dollars (USD), adjusting for inflation from the year in which the costs were incurred to the year 2015. We used the average exchange rate for the period May 2014 to January 2016 to convert values from FCFA (Central French African Francs) to USD (1 FCFA = USD 0.00164 in 2014; USD 0.00168 in 2015; USD 0.00167 in 2016).

In line with the conceptual approach described earlier, data collection reflected three steps: identification, measurement and valuation.

- Step 1: Identification

We started the data collection by asking the main stakeholders (MoH and WB) and implementing actors from the central level to share planning and implementation documents with us, including financial statements (e.g. initial budgets, project reports), that would allow us to reconstruct all activities carried out from the moment the intervention was conceived to the moment the identification cards were distributed to the ultra-poor. We examined all project documents in order to generate a detailed list of activities, including those of the main stakeholder as well as of the different implementation actors involved at each and every stage of the design and implementation phase (Table 1). This process allowed us to identify additional actors beyond those working directly for one of the agencies who had actively led design and/or implementation activities (such as development partners, academics, and consultants).

- Step 2: Measurement

**Table 1:** List of activities (aggregated)

List of Core Activities (aggregated)	Description
1. General Coordination/Management Design Phase	• All general activities performed during the design of the process (e.g. initial workshops to define the targeting strategy, seminars to develop the concept note, internal workshops hold by SERSAP
2. General Coordination/Management Implementation Phase	• All general coordination, management and supervision activities performed during the implementation phase (e.g. informational meetings at central, regional and district level and the national launch of the targeting intervention)
3. Training	• Training of ELMEO, CSS, GVL, CSI and the enumerators
4. Selection of the ultra-poor	• All activities related to the actual selection of in the villages (e.g. CSI meetings to select the ultra-poor and to establish lists, validation sessions by GVL to validate lists etc.)
5. Data Collection	• Development of the data collection program and questionnaires, preparation of tablets, data collection by 35 enumerators in the villages, photo taking and management of the ultra-poor database • Community members supported the enumerators to locate selected and assisted during data collection
6. Card Production/Distribution	• Every ultra-poor person received a personalized identity card to receive free services at health facilities • A Vietnamese IT company produced the ID cards and shipped them to Burkina Faso
7. M&E	• All monitoring and quality control activities

To estimate resource use, we triangulated information across data sources and filled gaps that emerged as we progressed through the data by conducting a series of face-to-face key informant interviews. We interviewed the two central level coordinators employed at SERSAP and the four regional coordinators. We used structured interview forms to ask respondents to recall the time spent by the various actors, including communities, on design and implementation activities (Additional files 2, 3 and 4). In addition, we asked key WB staff, academics, and consultants to estimate their time commitment to the program (interviews done by phone and/or email) (Additional file 5). WB staff was also instrumental in identifying material resource consumption, for instance in relation to the production of the cards.

- Step 3: Valuation

We first quantified the units of each resource and multiplied them by its unit costs. Reconstructing the intervention’s financial costs, for both personnel and material resources, was a relatively straightforward process, as financial transactions related to the intervention could easily be reconstructed by combining financial statements by SERSAP, PADS and the WB. Reconstructing the intervention’s economic costs was a more complex process. Material resources that could not be traced in the financial records, such as donated vehicles or donated supplies, were valued using current market prices, for instance average vehicle rental and average room rental prices. We used the human capital approach (according to which value of time is measured through the earnings of an individual [30] to value the time of personnel who contributed to design and/or implementation without being directly compensated for it. Specifically, we valued: 1. the time of MoH staff using average earnings for the different civil servant cadres [33]; 2. the time of community members using minimum daily wage (USD 2.31) [34]; 3. the time of all international development partners and consultants using standard WB consultancy rates (300 USD a day for a mid-career consultant and 600 USD a day for a senior consultant).

### Data analysis

First, keeping the differentiation between financial and economic costs and differentiating between the design and the implementation phase, we aggregated cost information by activity and by cost category. To simplify reading and facilitate understanding, we aggregated single activities into broad analysis categories by combining conceptual analogous activities. In addition, we aggregated costs by single actor by carefully assessing where expenses were really incurred. Then, we computed both financial and economic cost per ultra-poor person selected. As a final step, to test how the value of the intervention may change depending on variations in the cost of individual items, we conducted one-way sensitivity analyses, varying the percentage of overhead costs, the wage for informal workers and the budget provided by SERSAP including the costs for personnel, equipment, selection process and data collection. Additional file 6 provides an additional analysis of the financial and economic costs, first broken down by fixed and variables costs. The single activities with their costs are then assigned to the respective organizational level of the targeting program (national, regional, district, CSPS, village and indigent). The annex further provides cost functions which can be applied to estimate the total financial and economic costs for potential expansions of the program.

### Ethical considerations

Ethical clearance was granted by both the Ethics Committee of Heidelberg University (protocol S-272/2013) and by the Comité National d’Éthique pour la Recherche en Santé (CNERS) in Burkina Faso (protocol number 2013–7-066 and 2017–9-138). All parties linked to the intervention agreed on processing the data for this study. Information from informants was obtained anonymously.

## Results

Table 2 presents the financial and economic costs of the CBT intervention across activity clusters by phase. The estimated financial costs accounted for USD 587,510 and represent about 48% of the total economic value. The total economic cost of the CBT intervention was USD 1,213,447 The selection process represents the most relevant economic cost component. With an estimated economic value of USD 392,060 the selection of the ultra-poor carried out by community members represents one third of the total value of the intervention, followed by the data collection step with an estimated value of USD 328,958.

**Table 2:** Financial and economic costs by activity cluster (in USD)

Activity	Financial Costs	Percentage (%)	Economic Costs	Percentage (%)
Design Phase				
General Coordination & Management	6599	1%	62,174	5%
Implementation Phase				
General Coordination & Management	23,350	4%	63,177	5%
Training	86,298	15%	159,824	13%
Selection of the ultra-poor	85,162	14%	392,060	32%
Data Collection	187,948	32%	328,958	27%
Card Production & Distribution	107,000	18%	116,101	10%
M&E	11,339	2%	11,339	1%
Overhead (13% SERSAP, 20% WB)	79,814	14%	79,814	7%
Total	587,510	100%	1,213,447	100%

With a total of 102.609 identified ultra-poor persons, the financial costs amounted to USD 5,73 and the total economic costs to USD 11,83 per identified ultra-poor person.

### Distribution of financial and economic costs by actor

Sixty percent of the total financial costs (=USD 350,704) were incurred by activities carried out by the implementation agency. Forty percent of the financial costs (=USD 236,807) came about due to activities performed by the WB.

**Fig. 1:**
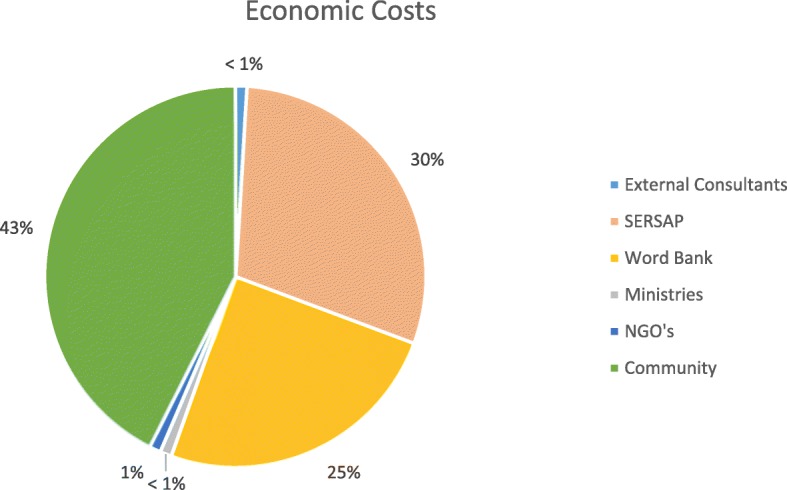
Distribution of economic costs by actor

Figure 1 shows the distribution of economic costs by actor. The highest proportion of economic costs were incurred at the community level (43%), followed by the implementation agency (30%) and the WB (25%). Only ≤1% of the economic costs was due to activities performed by external consultants, Non-governmental organizations (NGOs) and ministries.

### Financial and economic costs – Design phase by cost categories across activity clusters

Table 3 shows the financial and economic costs by cost categories for the activity “General Coordination & Management” performed at the design phase of the CBT intervention. The total amounted to USD 7457 financial costs and USD 63,032 economic costs, including the overhead costs. More than 80% of the financial costs and more than 70% of the economic costs were due to human resource expenses.

**Table 3:** Financial and economic costs – design phase by cost categories across activity clusters (in USD)

Activity Cluster	Financial Costs	Percentage (%)	Economic Costs	Percentage (%)
1. General Coordination & Management Design				
Human Resources	6348	85%	44,754	71%
Room Rental	0	0%	332	0.5%
Transportation	0	0%	16,837	26,7%
Consumables	251	3%	251	0.4%
Overhead	858	12%	858	1.4%
Grand Total	7457	100%	63,032	100%

### Financial and economic cost implementation phase by cost categories across activity clusters

**Fig. 2:**
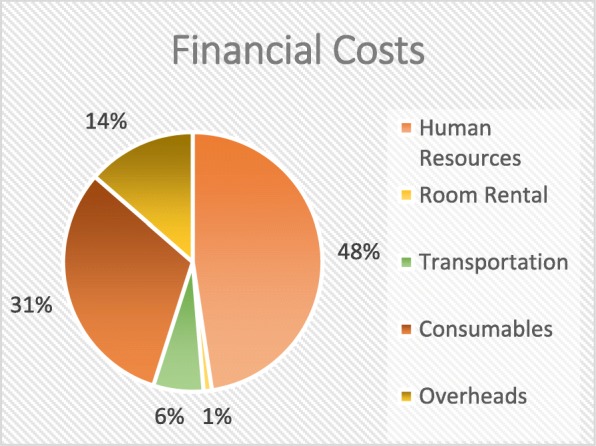
Distribution of financial costs – Implementation Phase

**Fig. 3:**
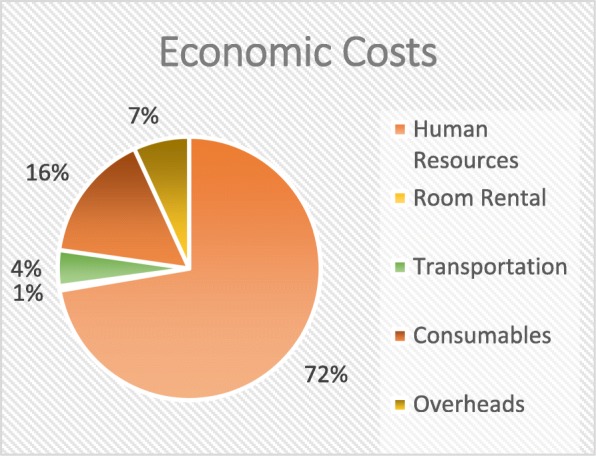
Distribution of economic costs – Implementation Phase

Table 4 shows the financial and total economic costs by cost categories across all activities performed during the implementation phase of the CBT intervention. The total amounted to USD 580,053 financial costs and USD 1,150,415 economic costs including the overhead costs. Just like in the design phase of the intervention, human resources accounted for the largest portion of implementation costs - almost 50% of the financial costs and 72% of the total economic costs (Figs. 2 and 3) . With USD 378,284, the biggest proportion of human resources was spent on the selection of ultra-poor, followed by the data collection with USD 274,601.

**Table 4:** Financial and economic costs – implementation phase by costs categories across activity clusters (in USD)

	Activity Cluster	Financial Costs (in USD)	Economic Costs (in USD)
(II) Implementation Phase	1. General Coordination & Management Implementation Phase		
	Human Resources	18,751	45,408
	Room Rental	126	358
	Transportation	2472	15,409
	Consumables	2002	2002
	Total	23,351	63,177
	2. Training		
	Human Resources	44,408	117,895
	Room Rental	5862	5862
	Transportation	11,218	11,218
	Consumables	24,809	24,849
	Total	86,297	159,824
	3. Selection of the ultra-poor		
	Human Resources	71,386	378,284
	Room Rental	0	0
	Transportation	12,947.	12,947
	Consumables	829	829
	Total	85,162	392,060
	4. Data Collection		
	Human Resources	133,591	274,601
	Room Rental	0	0
	Transportation	6463	6463
	Consumables	47,894	47.894
	Total	187,948	328,958
	5. Card Production/Distribution		
	Human Resources	0	7565
	Room Rental	0	0
	Transportation	0	1536
	Consumables	107,000	107,000
	Total	107,000	116,101
	6. M&E		
	Human Resources	8140	8140
	Room Rental	0	0
	Transportation	3199	3199
	Consumables	0	0
	Total	11,339	11,339
	Overhead	78,956	78,956
	Grand Total Implementation Phase	580,053	1,150,415

### Distribution of financial and economic costs - implementation phase

Figures 2 and 3.

#### Sensitivity analysis

The sensitivity analysis revealed that varying the wage for informal workers from minimum to average wage did not have a major impact on the total economic costs. By applying this variation, the total economic costs only changed by USD 50,506 (= 4.2%) and amounted to USD 1,263,953 (Additional file 7). Varying the percentage of overhead costs also only had little impact on the total costs of the CBT intervention. More specifically, increasing the percentage of overhead costs (16% for SERSAP and 22% for WB) led to an increase by USD 13,258 and amounted to a new economic value of USD 1,226,705. On the other hand, reducing the percentage of overhead costs (10% for SERSAP and 18% for WB) resulted in a new economic value of USD 1,200,190 (Additional file 8). Varying the budget provided by SERSAP also impacted the overall economic value only marginally. Increasing the SERSAP budget (costs for personnel, equipment and the selection process) by 5% amounts to economic costs of USD 1,230,983 by 10% to USD 1,248,517; by 15% to USD 1,266,051 and by 20% to USD 1,283,550. Decreasing the SERSAP budget by 5% amounts to economic costs of USD 1,195,911; by 10% to USD 1,178,377; by 15% to USD 1,160,840; and by 20% to USD 1,143,306 (Additional file 9).

## Discussion

This study is first of its kind to accurately trace both the financial and economic costs of CBT. By assessing the overall economic value of a CBT intervention, our study makes a unique contribution to the very limited literature available on the cost of targeting in general, both within and beyond the health sector. The lack of comparable empirical literature seems to be due to the fact that the budget statements of targeting interventions in LMICs do not generally indicate costs by process or activity [11]. Lack of comparable studies limits our ability to discuss our results extensively in light of prior evidence, especially when considering the economic value of the intervention. Nevertheless, when relevant, we do appraise our findings in relation to the two published studies that assessed the financial costs of CBT [18, 19] and to the one unpublished report [11] which also accounted for the value of the time contributed by uncompensated community members.

A unique trait of our study is its reliance on ABC, a conceptual approach identified as the preferred one to cost complex health interventions, also in LMICs [35]. The application of ABC allowed us to estimate both the financial and economic costs of the CBT intervention per activity in a highly systematic and accurate manner. This high level of accuracy was ensured by the fact that instead of relying on financial statements as our primary source of information, we started our work by tracing all activities that made up the intervention and all resources consumed by these activities, looking for corresponding financial values only afterwards. Furthermore, combining ABC with Resource Consumption Accounting [36] allowed us to trace the single cost categories within each activity, generating a very detailed map for policy makers of what activities and what items within activities drove the intervention costs. It needs to be noted, however, that the application of this methodological approach is usually not inexpensive [31]. In our specific case, we could rely on ABC costing only because our access to all implementation documents was facilitated by our team’s close relation with the implementation teams and its development partners.

We estimated a financial cost of USD 587,510 and an economic cost of USD 1,213,447 for the entire CBT intervention, including its design and implementation phase. With a total of 102,609 identified ultra-poor, this corresponds to a financial cost of USD 5.73 and an economic cost of USD 11.83 per ultra-poor person identified. As demonstrated in Additional files 2, 3 and 4, our results were neither highly sensitive to variation of the applied wage nor to changes in the percentage of overhead costs or the budget provided by SERSAP. The consistency of our results from primary analysis with those of the sensitivity analysis suggests a good robustness of our findings.

Our study estimated financial costs per ultra-poor person identified to be within the same range of what was estimated in the few prior comparable studies. In particular, Ridde et al. [18], traced the financial expenses of a CBT action research project in one district of Burkina Faso and calculated financial cost of USD 10.16 per identified person. The financial costs Aryeetey et al. [19] calculated for a Participatory Wealth Ranking exercise in Ghana were calculated to be USD 3.83 per identified person in an urban setting, USD 10.63 for a rural setting and 2.71 for a semi-urban setting (calculation: survey costs without exemption premium divided by the total number of people exempted). Compared to the study of Watkins [11], who calculated financial cost of approximately USD 25 per recipient, our estimate is much lower. Differences are likely to be attributable to different implementation strategies as well to differences in methodological pathways to cost estimation.

Additionally, the study draws attention to the large discrepancy between financial and economic cost.Similarly to Watkins [11], we found the difference to be driven by the valuation of human resources, specifically the fact that we valued the time contributed towards the initiative by community volunteers. The heavy reliance on communities as key players in the implementation of the targeting intervention is well aligned with the push towards decentralization and community involvement, which has prevailed across sub-Saharan Africa following the Bamako Initiative [37]. Still this high reliance on communities as actual implementing agents raises important questions in terms of acceptability and feasibility. On the one hand, targeting of health services based on community-based approaches is likely to result in a more accurate and more acceptable identification strategy than one based on experts’ (i.e. healthcare providers) judgment [9, 16, 28, 38] and is likely to be more attractive in the eyes of implementing agencies given its relatively low financial costs [11]. On the other hand, our study indicates that the time committed by volunteers has a clear opportunity cost, measured in terms of their foregone economic value. This observation questions the adequacy of executing CBT by adding an additional burden to communities already operating in resource-constrained settings and often struggling to meet their most basic needs [16, 38]. The challenge is that of relying on the expertise of communities, while at the same time offering adequate compensation for their engagement [39]. This is meant to sustain their motivation in contributing to such activities and avoid withdrawal during the implementation phase, as observed in some communities during the intervention described in our study [29].

Looking at the distribution of costs across activities provides additional evidence that financial and economic valuations do not necessarily align, as we see that the actual identification of the ultra-poor (the core activity delegated to communities) accounted for less than one sixth of all financial costs, but for one third of all economic costs. Moreover, the high cost associated with preparing for and producing identification cards may be surprising for those unfamiliar with the intervention, since the two activities combined accounted for nearly 50% of all financial costs. While we have been reassured by key stakeholders (personal communication) that the cost of card production was much lower in Vietnam compared to what it would have been in Burkina Faso, our appraisal of this specific set of findings inevitably questions the need for sophisticated individual digitalized cards vs. simpler household traditional paper-based cards. It is beyond the scope of our work to assess the comparative advantage of a system based on digitalized vs. traditional paper-based identification cards, albeit we do imagine this to be a relevant area for further research, estimating cost-effectiveness of one system compared to the other. Likewise, the government and its partners, in Burkina as elsewhere, could test the possibility of lowering CBT costs by integrating activities related to the database establishment into the initial identification effort.

Furthermore, when assessing both the financial and the economic value of targeting interventions, one must consider the extent to which these are driven by national governments or by development partners [40]. In the specific case analyzed in our study, the largest portion of the financial costs (60%), excluding the card production that was paid directly by the WB, were accrued by a local for-profit organization, SERSAP, which in turn was contracted by the governmental agency PADS. In practice, however, the resources deployed for the identification and selection procedure originated in a grant made by the HRITF to the government of Burkina Faso, channeled via the PADS. This explicit donor involvement sheds yet a different light on the appraisal of the financial costs associated with targeting programs. Spending close to USD 6 per ultra-poor person identified may be affordable for international agencies specifically committed to fostering greater equity in access, but is likely to be unaffordable for most LMICs, considering overall low levels of public spending on health [41]. Specifically, without aiming at conducting a full budget impact analysis – a task beyond our initial study objectives – it is worthwhile to appraise financial costs in relation to the overall health budget of Burkina Faso. The USD 5.73 per ultra-poor person identified are equivalent to 21% of Burkina Faso’s per capita government expenditure on health (USD 27 (in PPP International USD, year: 2015)) [42]. At the same time, however, one must consider that targeting approaches are implemented beyond the health sector alone and hence there may be potential in sharing costs for pro-poor identification and selection procedures across sectors [43, 44].

### Methodological considerations

We also faced some challenges during data collection and analysis, which are worthwhile discussing here as potential limitations of our study. First, we assessed the value of resources consumed by SERSAP activities on the basis of the unit costs and the units consumed as indicated in the initial implementation budgets rather than in the closing financial statements, to which we could not gain access. Hence, we cannot fully exclude having over or underestimated unit costs or resource consumption for some items. Nevertheless, given that we triangulated information coming from SERSAP documentation with information obtained during the key informant interviews, we are quite confident that we have not largely misestimated these values. Second, our analysis did not account for any fixed costs incurred at SERSAP as the agency was already operative and the targeting process only built upon it. This might have led to an underestimation of the full cost of CBT. Third, we must also mention that tracing all activities and all related resource consumption was difficult due to the retrospective nature of our study. It would have been desirable to conduct a prospective study which could have generated more accurate data [35] by applying the ABC-method from the onset. The retrospective nature of the study also opened the way to recall bias, since we interviewed key players about one year after cards were distributed.

## Conclusion

Universal Health Coverage can only be achieved by ensuring that the poorest are not left behind. Thus, it is necessary that once implemented, measures to identify and address health inequity are systematically evaluated. With our study, we set the first example of how to systematically assess both the financial and economic costs of a community-based targeting program in a LMIC setting. We view the contribution of this costing study as meaningful, particularly because pro-poor targeting programs continue to expand, within and beyond the health sector. Future research should not only replicate this approach in other settings to generate additional and comparable evidence to better inform policy, but should also reach beyond the mere estimation of the costs to assess the costs of targeting in relation to the benefits accrued, within the framework of a comprehensive economic evaluation, such as a cost-effectiveness analysis.

## Additional files

Additional file 1: Implementing actors with level, composition and responsibilities. (DOCX 16 kb)

Additional file 2: Data Collection Form SERSAP. (DOCX 1872 kb)

Additional file 3: Data Collection Form Regional Technical Assistants (ATR’s). (DOCX 2353 kb)

Additional file 4: Self-administered Questionnaire WB. (DOCX 288 kb)

Additional file 5: Interview Guide. (DOCX 519 kb)

Additional file 6: Financial and economic costs in USD differentiated between fixed and variable costs and organizational level. (DOCX 24 kb)

Additional file 7: Sensitivity Analysis: Wage Informal Workers (in USD). (DOCX 14 kb)

Additional file 8: Sensitivity Analysis: Increase and Decrease of overhead costs (in USD). (DOCX 16 kb)

Additional file 9: Sensitivity Analysis: Increase and decrease SERSAP Budget (in USD). (DOCX 15 kb)

## Abbreviations

ABC: Activity-based costing
ATRs: Assistant Technique Régional (Regional Technical Assistants)
CBT: Community-based targeting
CNERS: Comité National d’Ethique pour la Recherche en Santé́ (Ethical Committee Burkina Faso)
COGES: Comité de gestion (Management Committee)
CSI: Comité de suivi de la sélection/cellule de sélection des indigents (Community Selection Committee)
CSPS: Centre de Santé et de Promotion Sociale (Primary Health Care Centers)
CSS: Comité de suivi de la sélection (Selection Monitoring Committee)
ELMEO: Equipe Nationale de Mise En Œuvre/de coordination (Local Implementation Team)
FCFA: Franc of the communauté financière Africaine (CFAF: Central French African Francs)
GVL: Groupe de validation locale (Local Validation Group)
HRITF: Health results innovation trust fund
ICP: Infirmier chef de poste (Head nurse)
LMICs: Low and middle income countries
M&E: Monitoring and evaluation
MoH: Ministry of health
MT: Means-test
NGOs: Non-governmental organizations
PADS: Programme d’Appui Au Développement Sanitaire (Health Development Support Program)
PBF: Performance-based financing
PMT: Proxy means testing
SERSAP: Société d’Etude et de la Recherche en Santé Publique (Society for Studies and Public Health Research), Burkina Faso
USD: US-dollar
WB: World bank
WHO: World Health Organization
