# Supplementary material for: How much does community-based targeting of the ultra-poor in the health sector cost? Novel evidence from Burkina Faso
Source: Health Econ Rev. 2018 Sep 4;8:19. doi: 10.1186/s13561-018-0205-7 (PMC6123332; doi:10.1186/s13561-018-0205-7)
Supplement: Supplementary file 1 — Implementing actors with level, composition and responsibilities. (DOCX 16 kb) [file 13561_2018_205_MOESM1_ESM.docx]

**Additional file 1: Implementing actors with level, composition and responsibilities**

| LEVEL | ACTOR | Members | Responsibilities |
| --- | --- | --- | --- |
| International Level | WB | 3 Consultants | To coordinate and manage the CBT intervention at the international level e.g. procurement of devices; development and management of indigent database; field supervision |
|  | External Consultants | Researchers from Heidelberg University and University of Montreal | To consult the design of the targeting intervention |
| National Level | Ministry of Health’s Technical Support Unit | Multiple members | To officially oversee the implementation of the entire targeting intervention |
|  | National Implementation Team (SERSAP) | 1 Technical Coordinator  1 M&E Manager  1 Financial Officer  2 Short term staff | To oversee, supervise and manage the entire CBT intervention at the national level |
| Regional Level | Technical Assistants per Region (ATRs) | 1 Technical Assistant per Region | To ensure the implementation of the CBT intervention at the regional level |
| District Level | Local Implementation Team (ELMEO) | 2 Members of the district management team  1 Agent of the provincial directorate of social action | To ensure the implementation of the CBT intervention at the district level |
| CSPS Level | Selection Monitoring Committee (CSS) | 2 COGES Members  1 Nursing Chief  1 Village Councillor  1 Councillor from CSPS headquarter | To monitor the selection of the ultra-poor at the village level |
|  | ICP and Health Workers | 7 ICP members/health workers | To facilitate the selection process in the CSPS area |
| Village Level | Community Selection Committee (CSI) | 7 Community members  *gender-balanced* | To ensure the selection of the ultra-poor in the villages |
|  | Local Validation Group (GVL) | 1 Chief or traditional leader  1 Religious Leader  1 President of CVD  1 Second councillor of the area | To internally validate the selection of the ultra-poor |
