# Supplementary figures and images for: How much does community-based targeting of the ultra-poor in the health sector cost? Novel evidence from Burkina Faso
Source: Health Econ Rev. 2018 Sep 4;8:19. doi: 10.1186/s13561-018-0205-7 (PMC6123332; doi:10.1186/s13561-018-0205-7)

## **Additional file 2: Data Collection Form SERSAP**


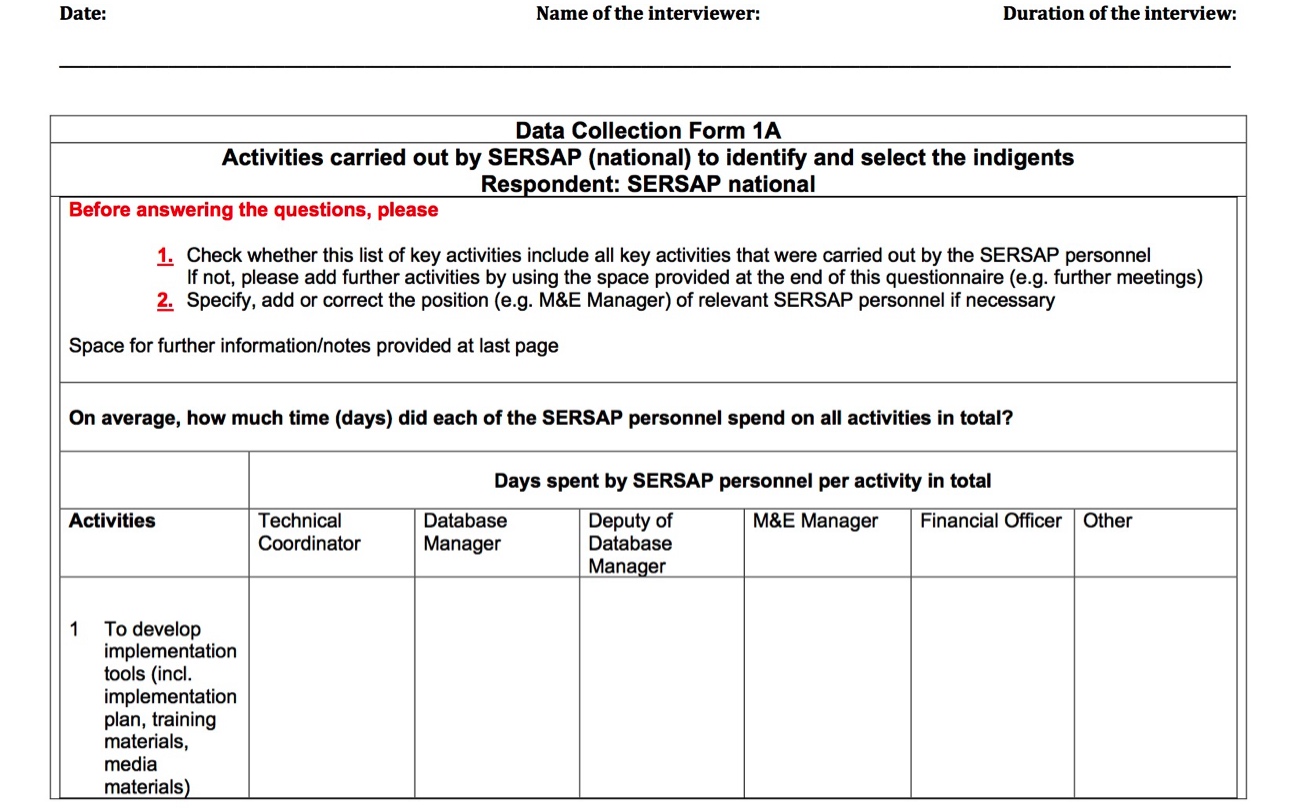


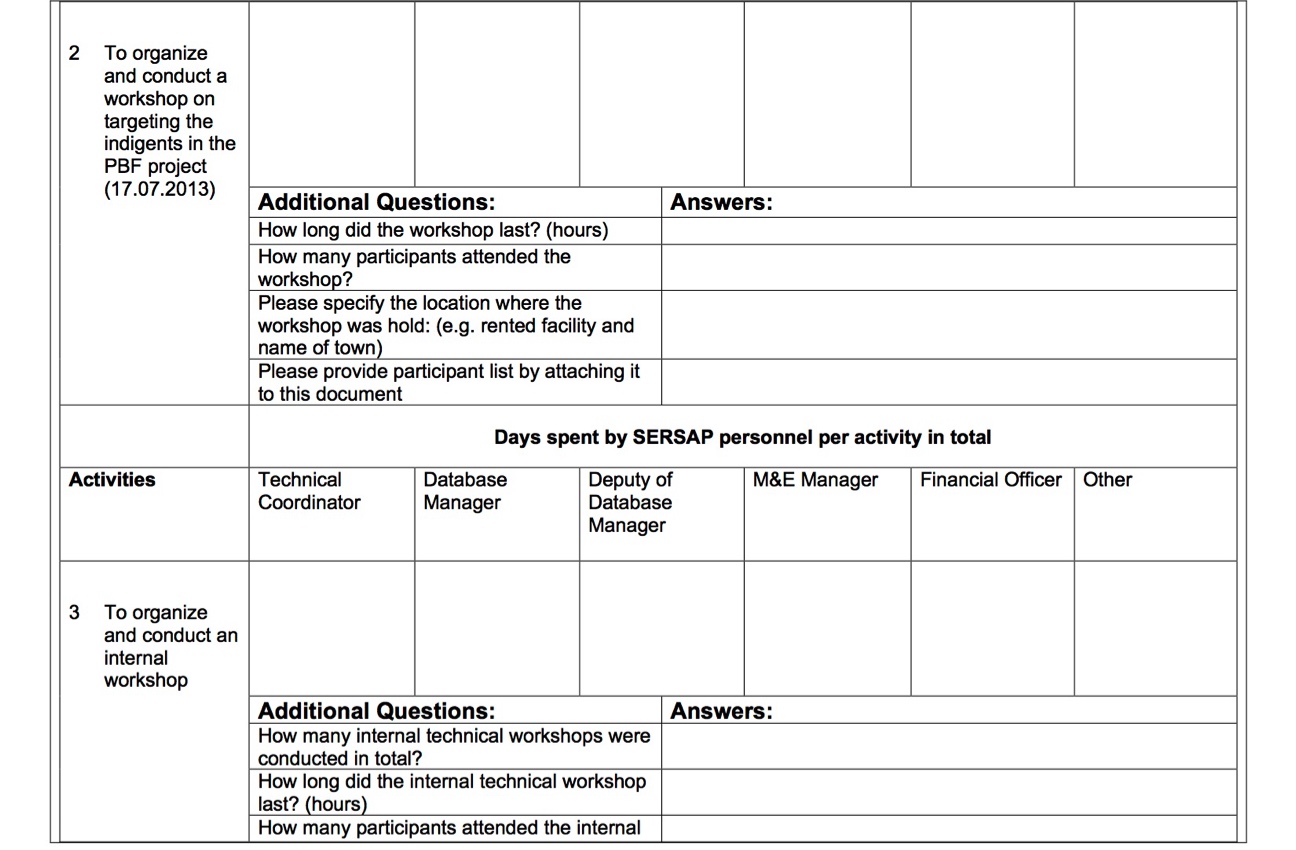


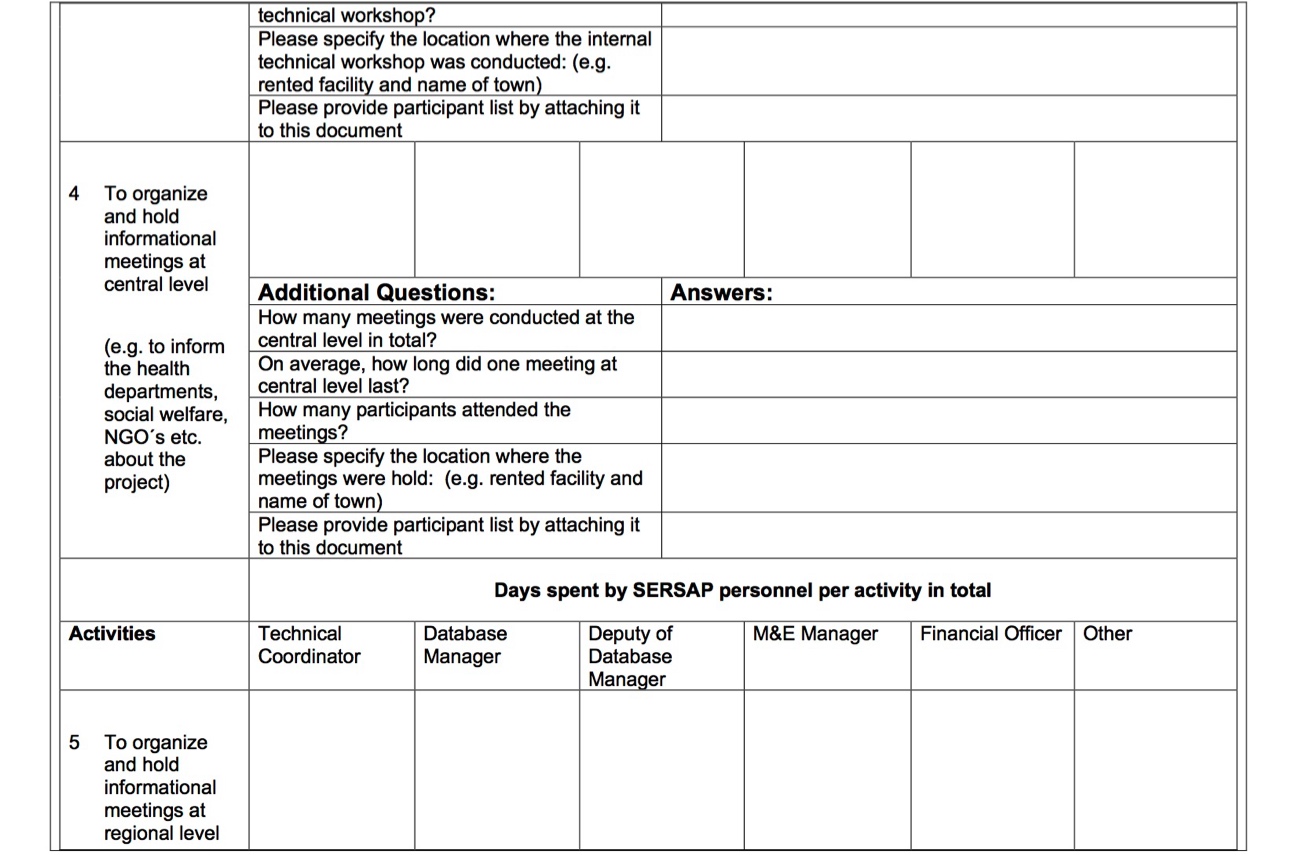


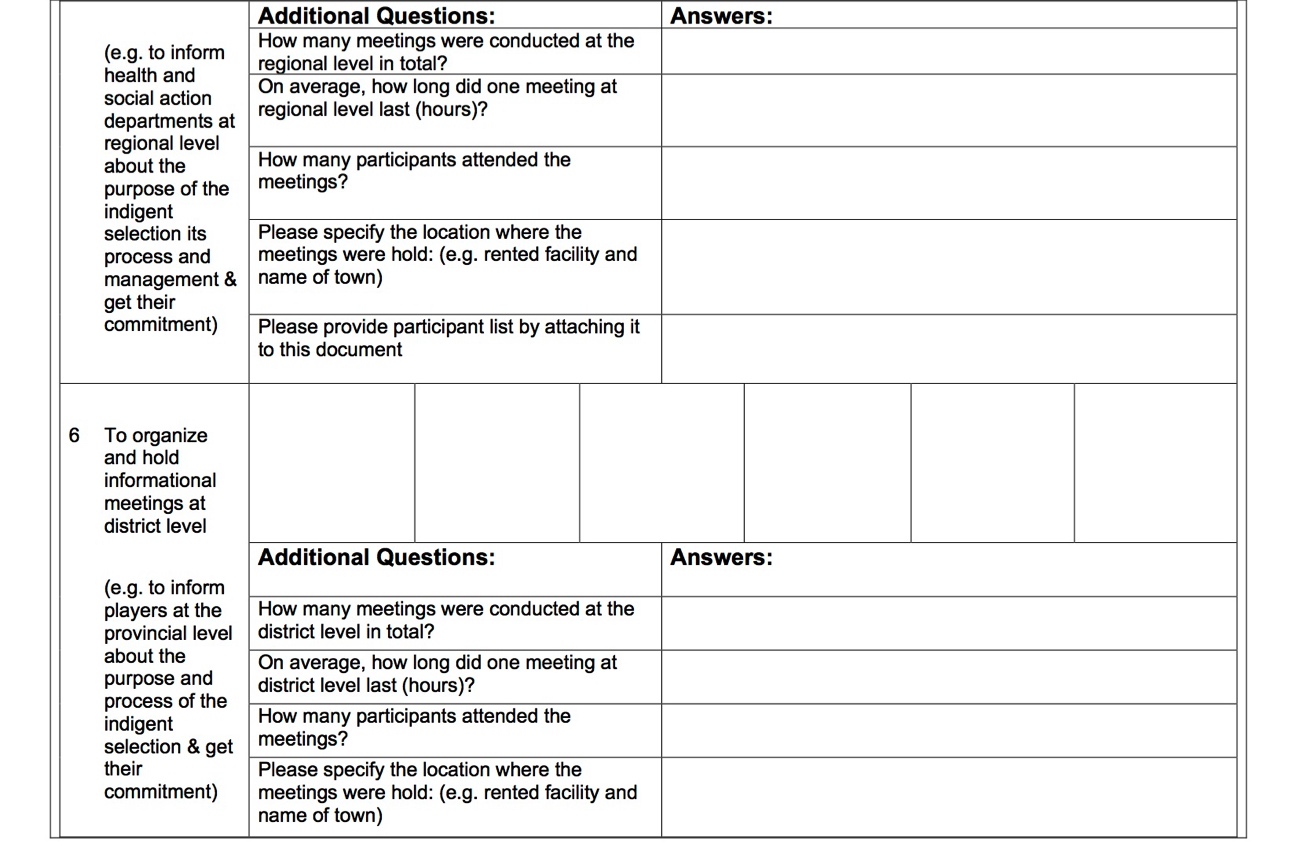


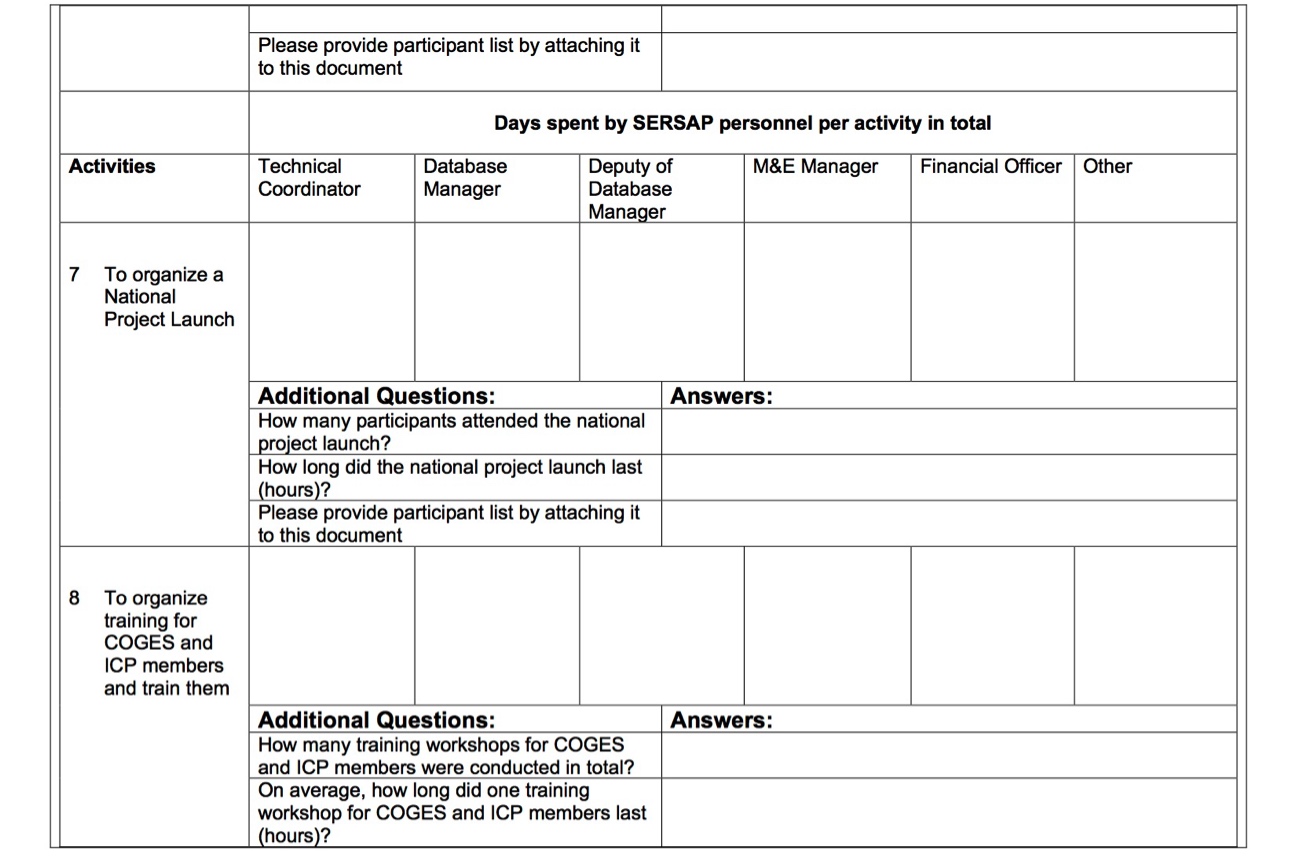


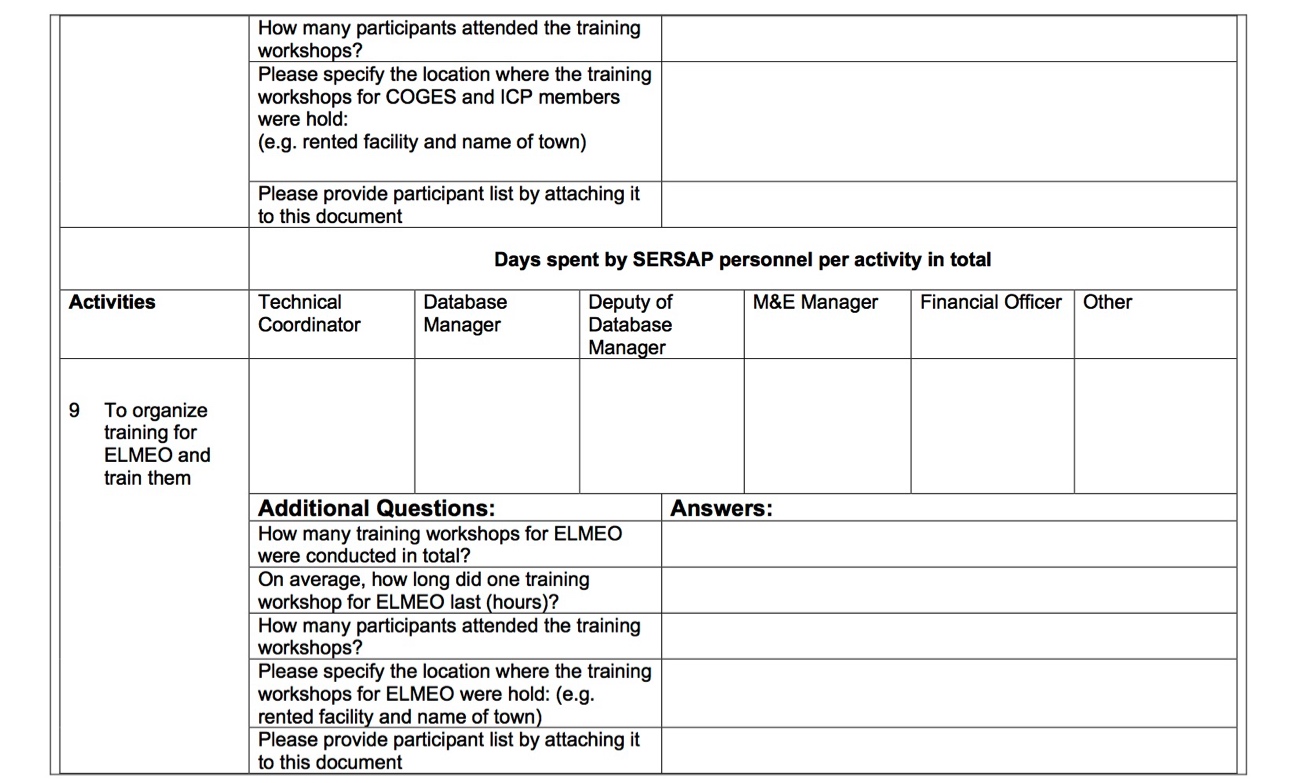


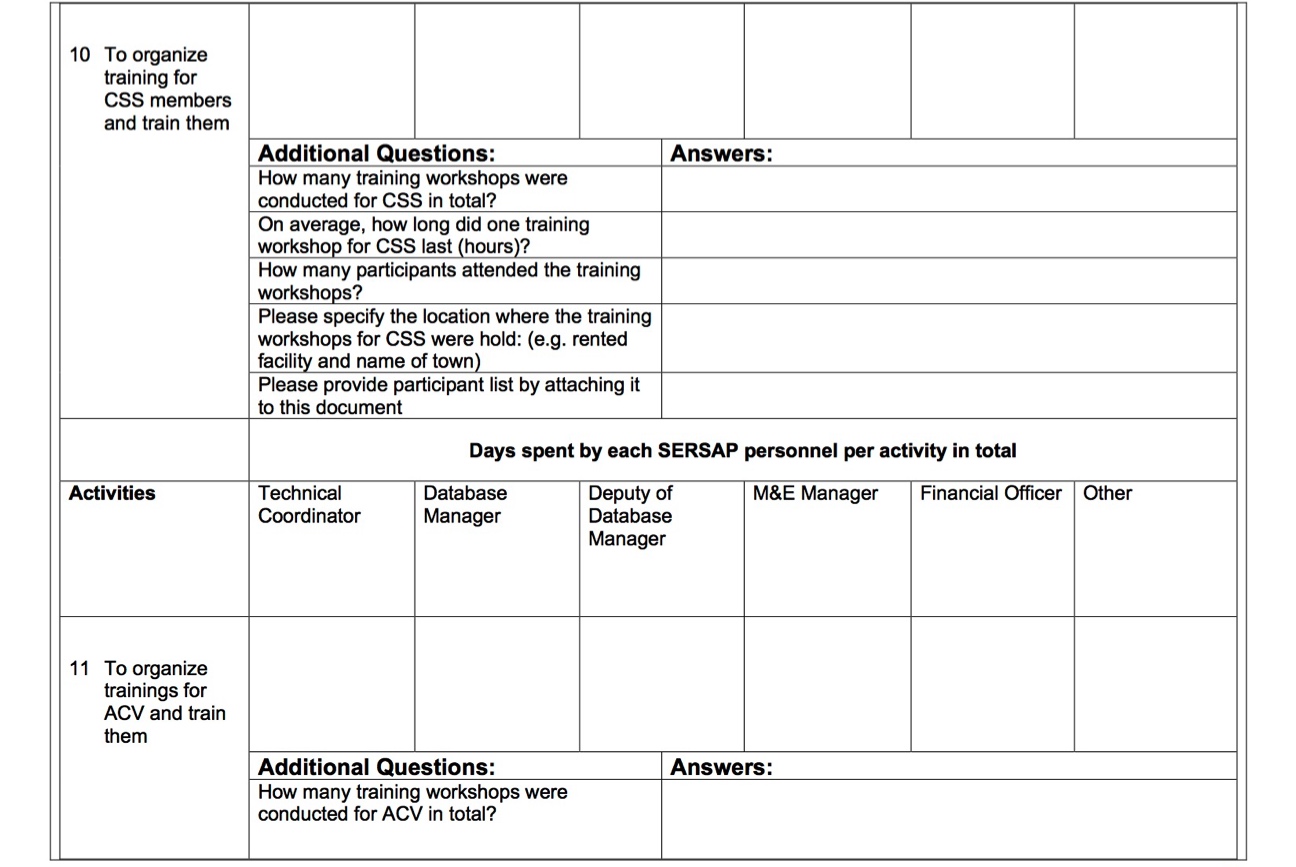


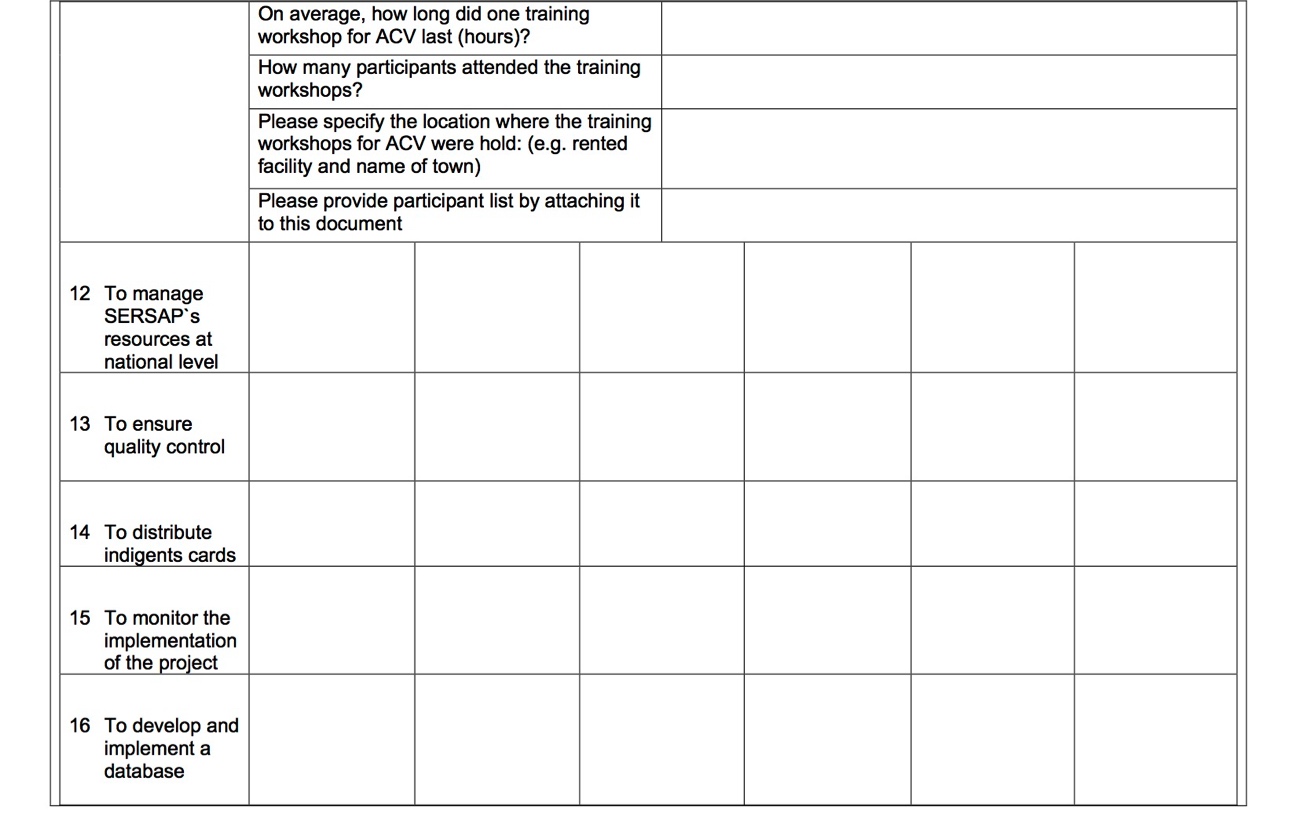


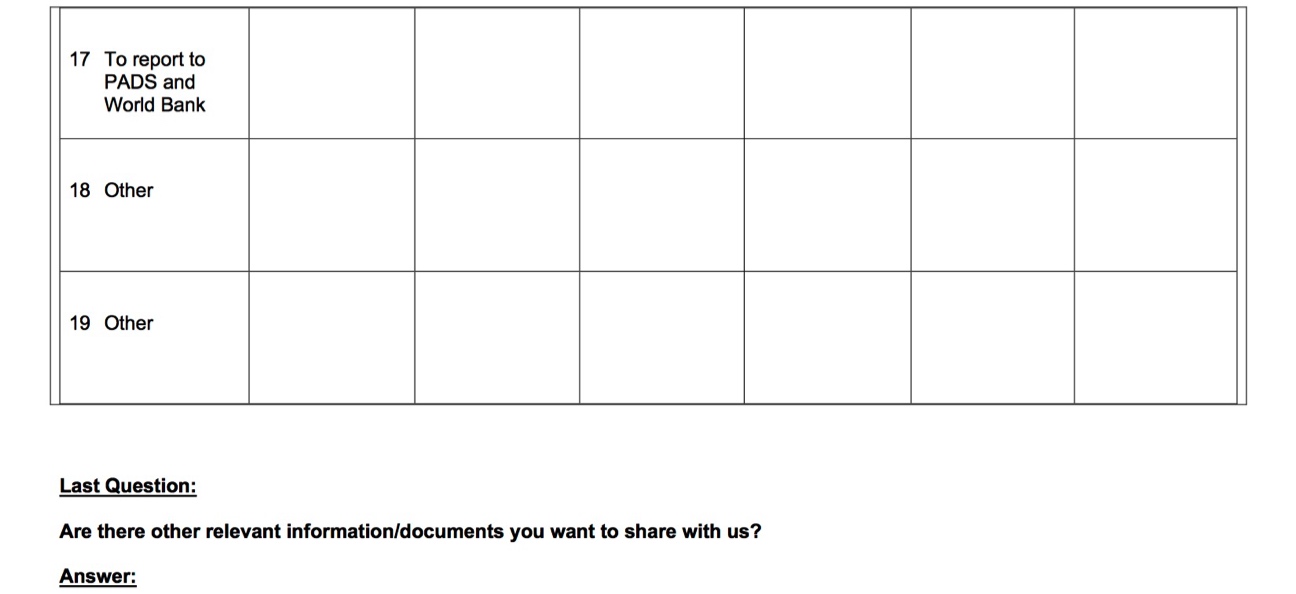

Supplement: Supplementary file 2 — Data Collection Form SERSAP. (DOCX 1872 kb) [file 13561_2018_205_MOESM2_ESM.docx]

## **Additional file 3: Data Collection Form Regional Technical Assistants (ATR´s)**

**
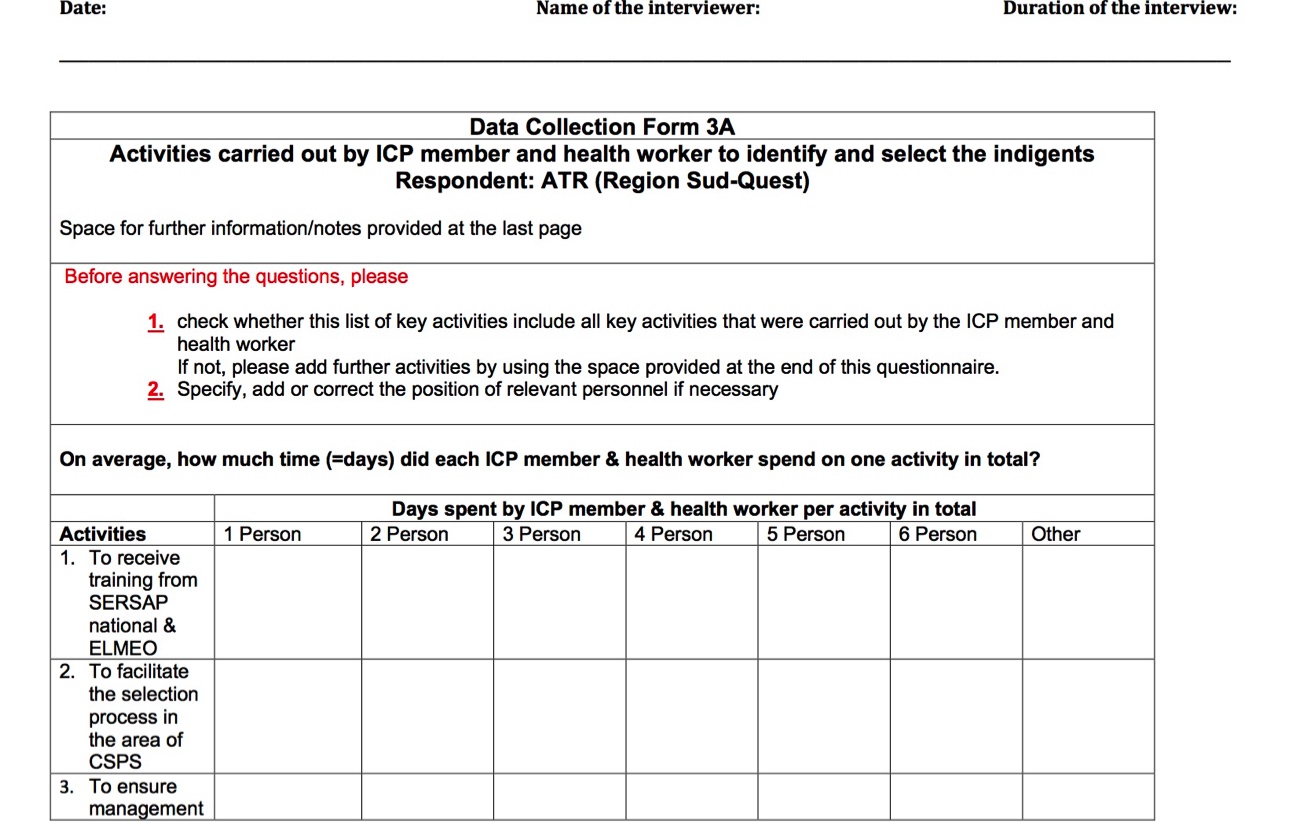
**


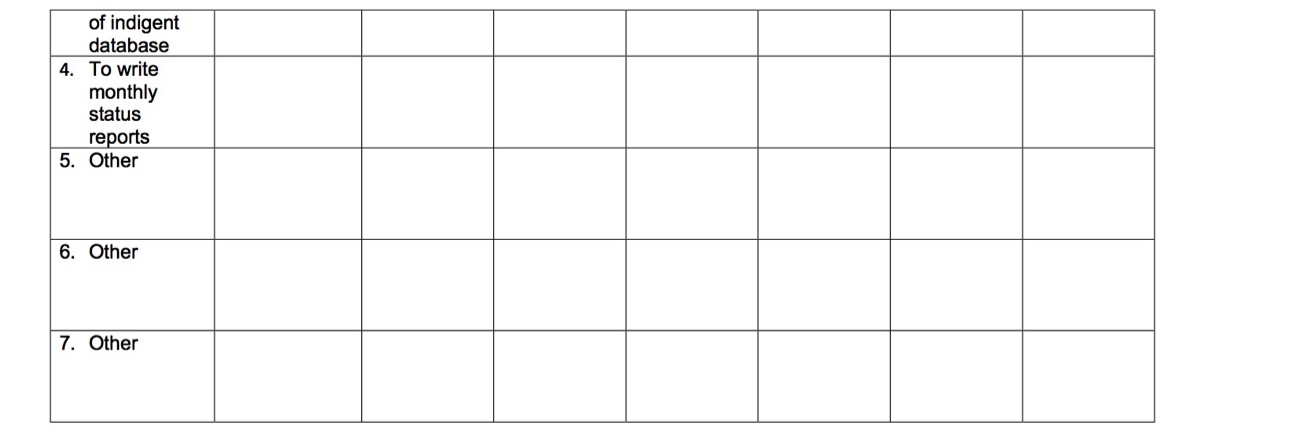


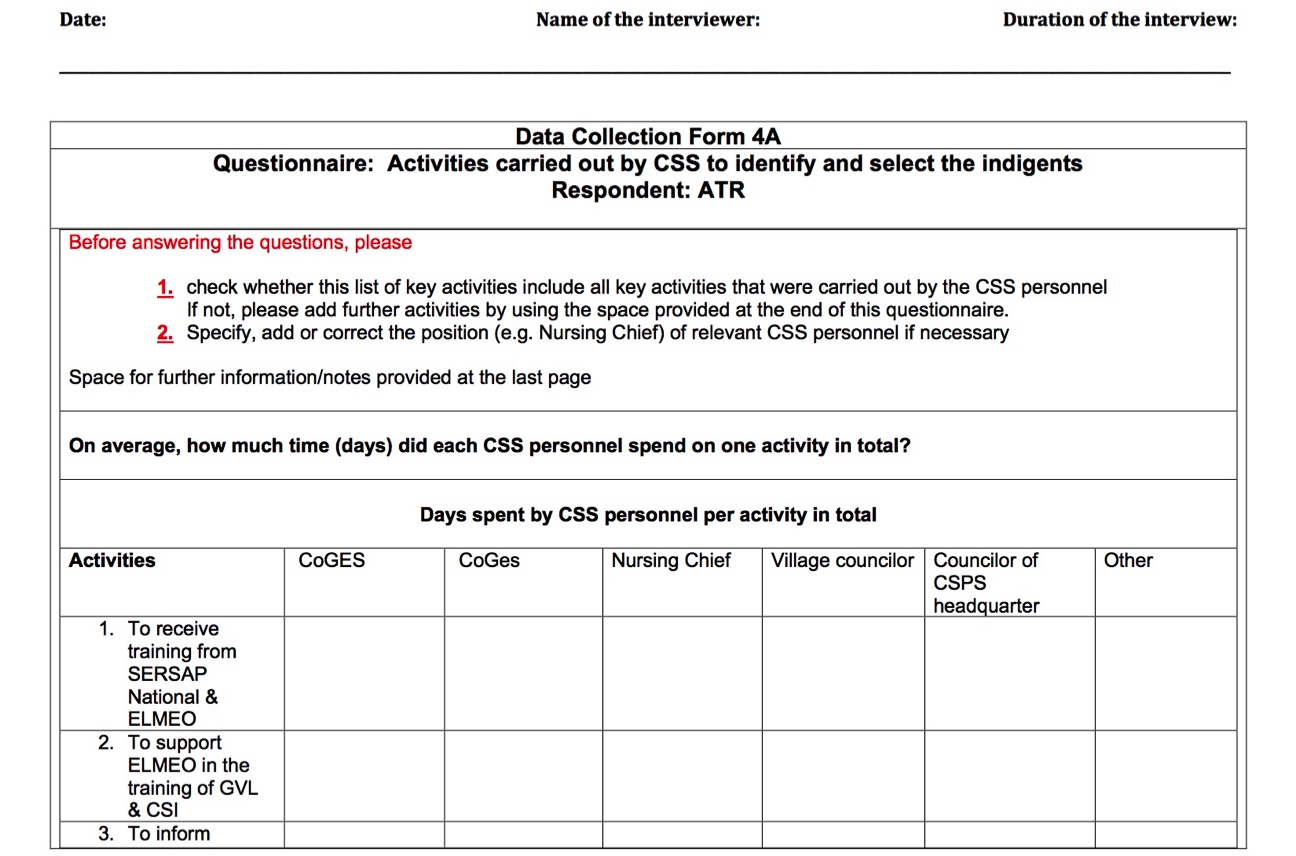


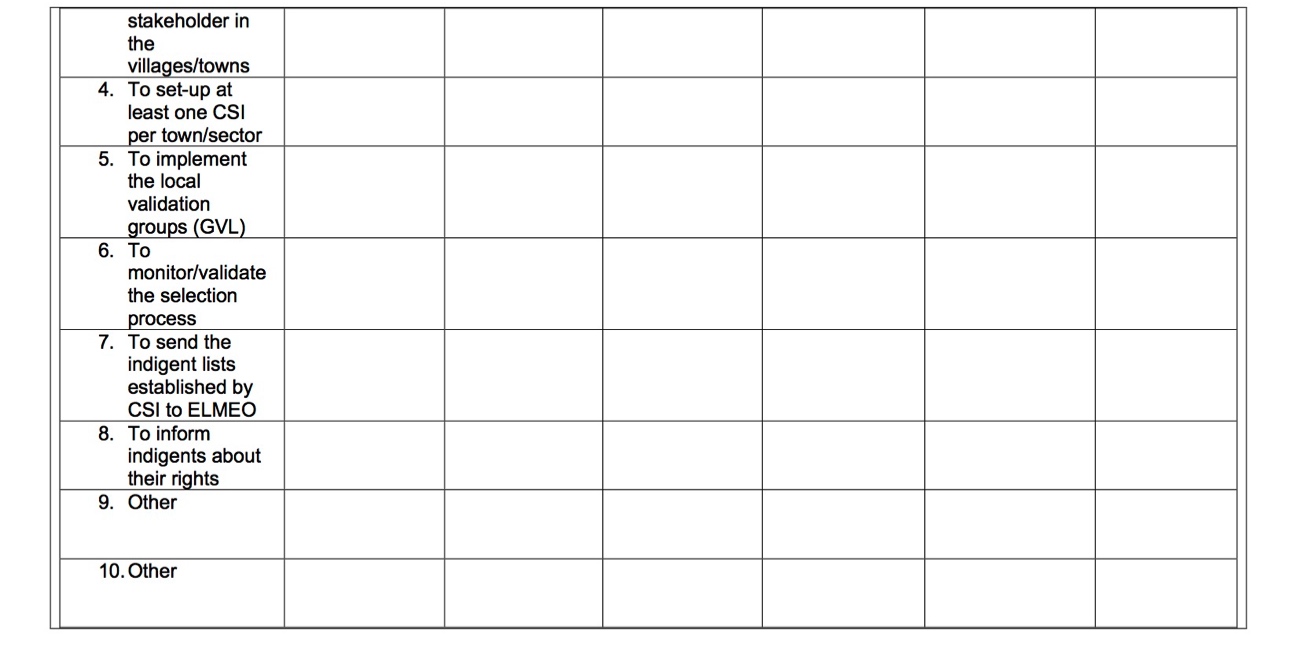


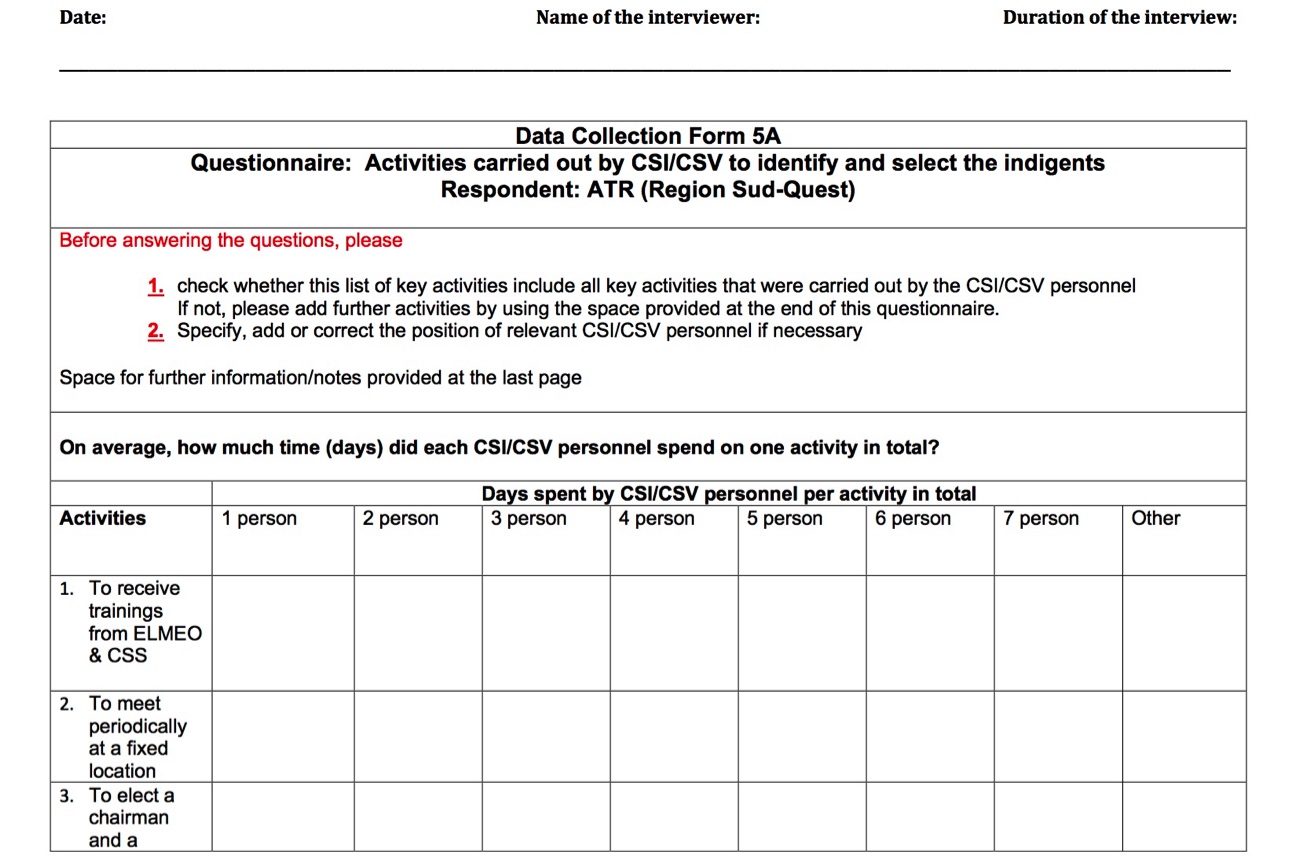


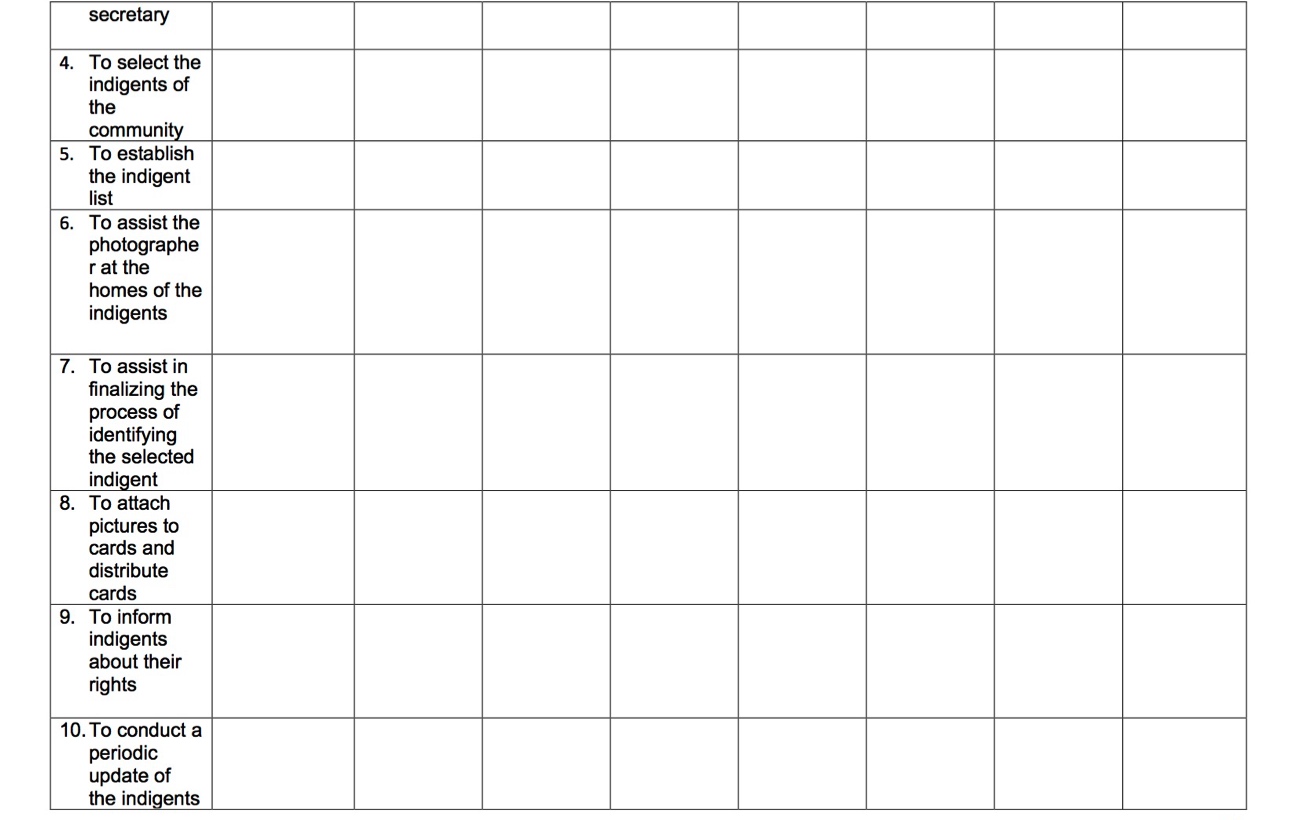


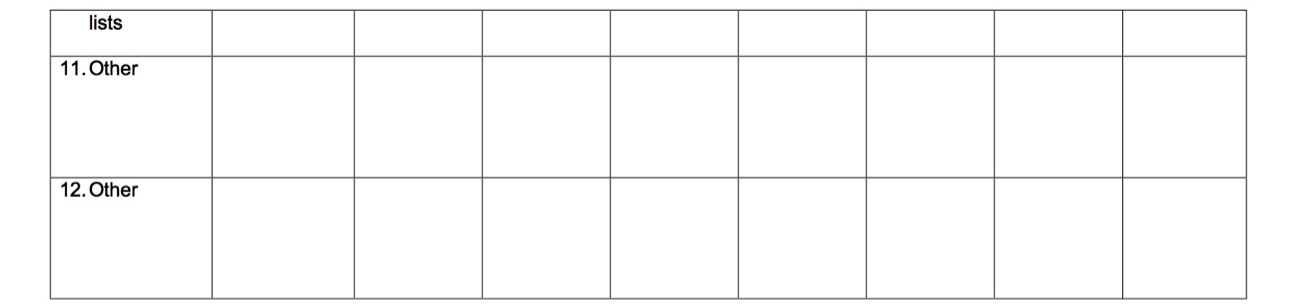


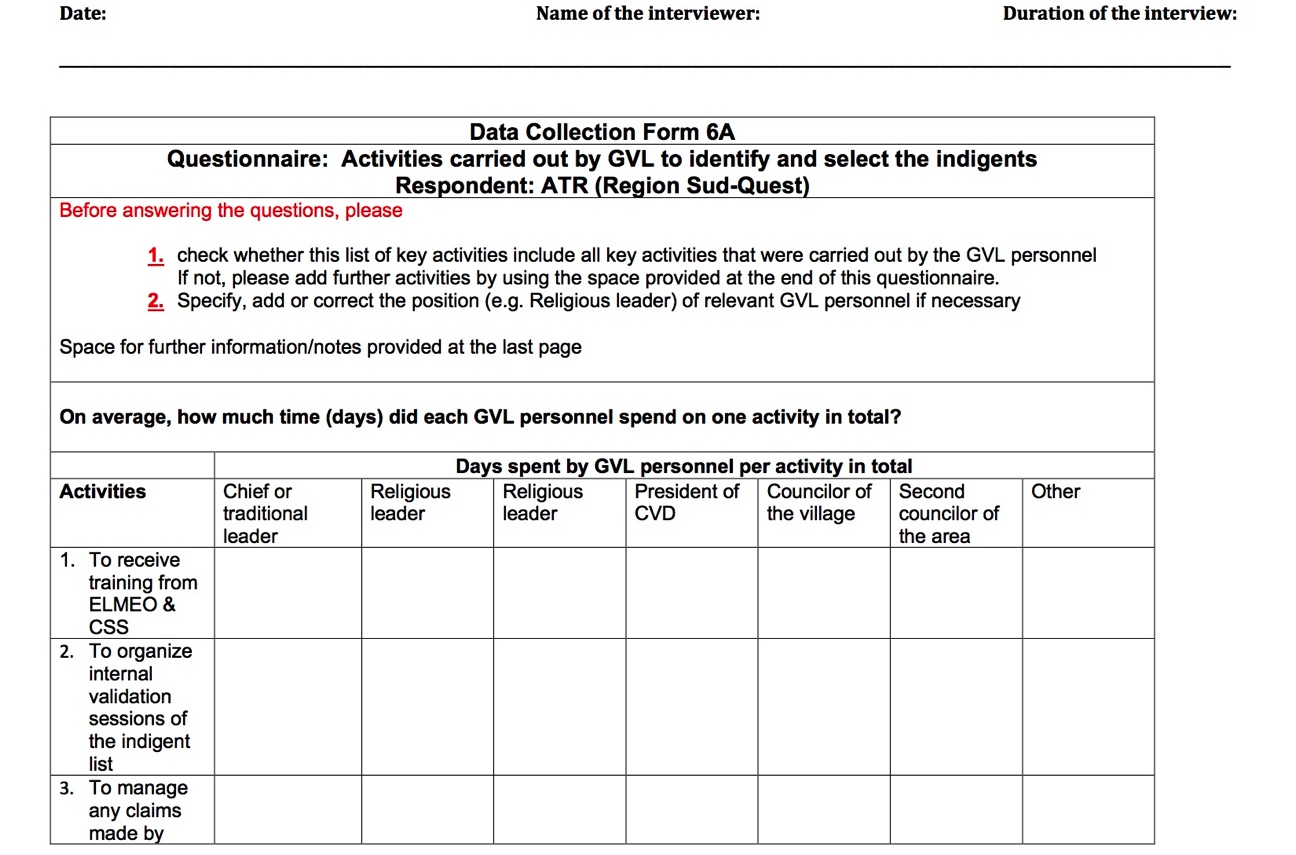


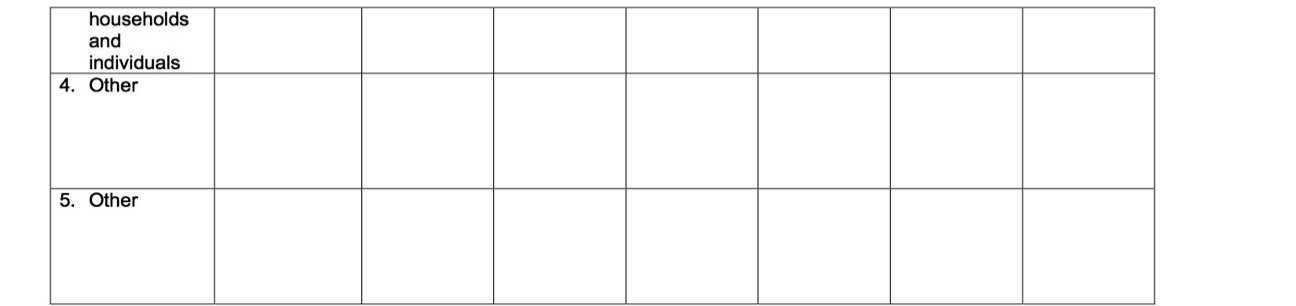


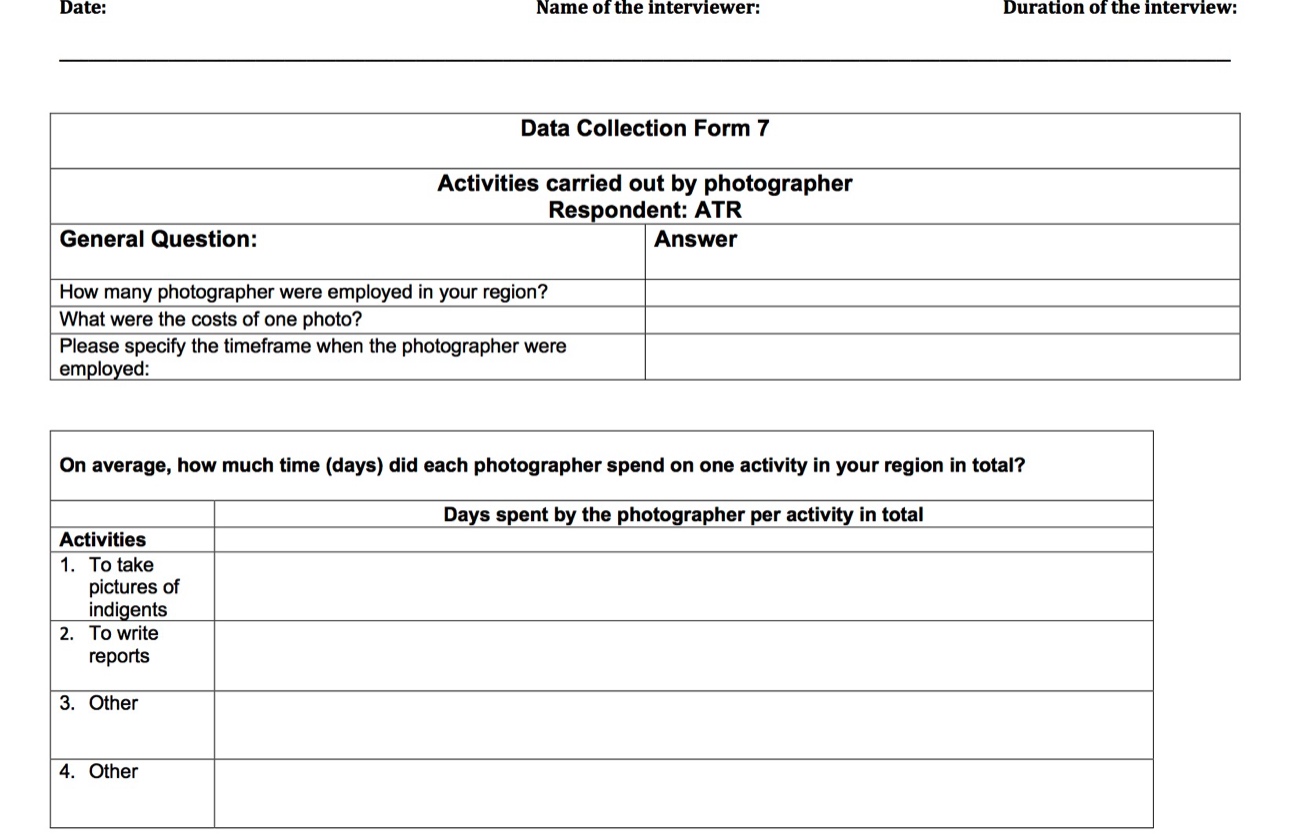


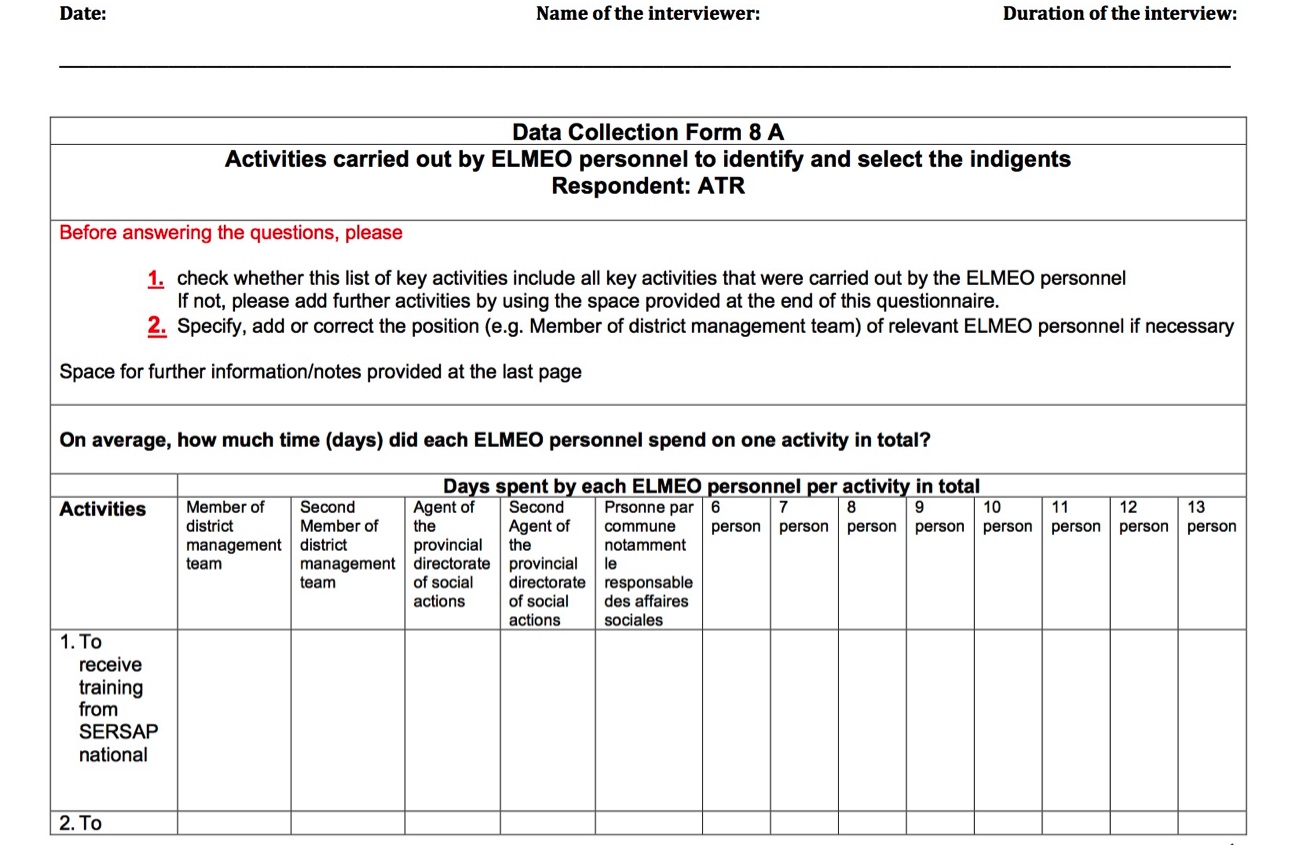


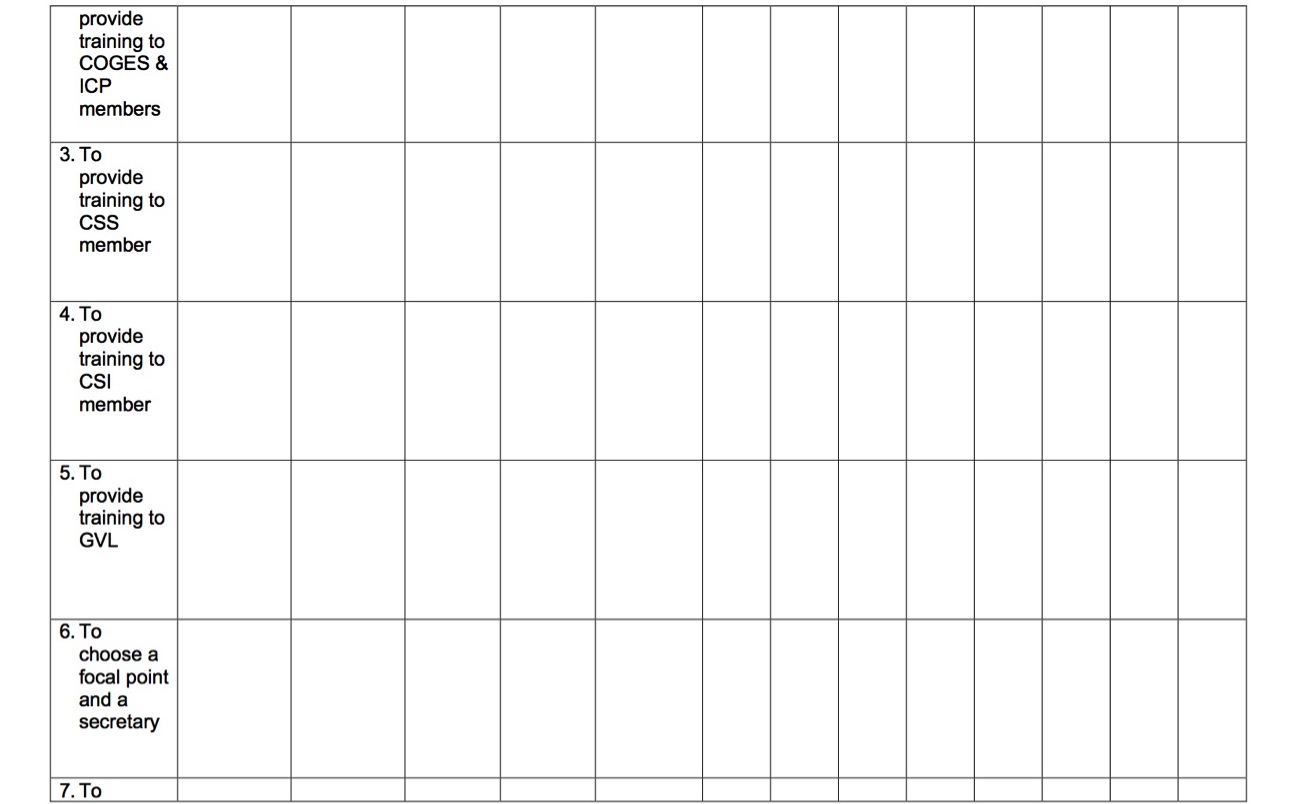


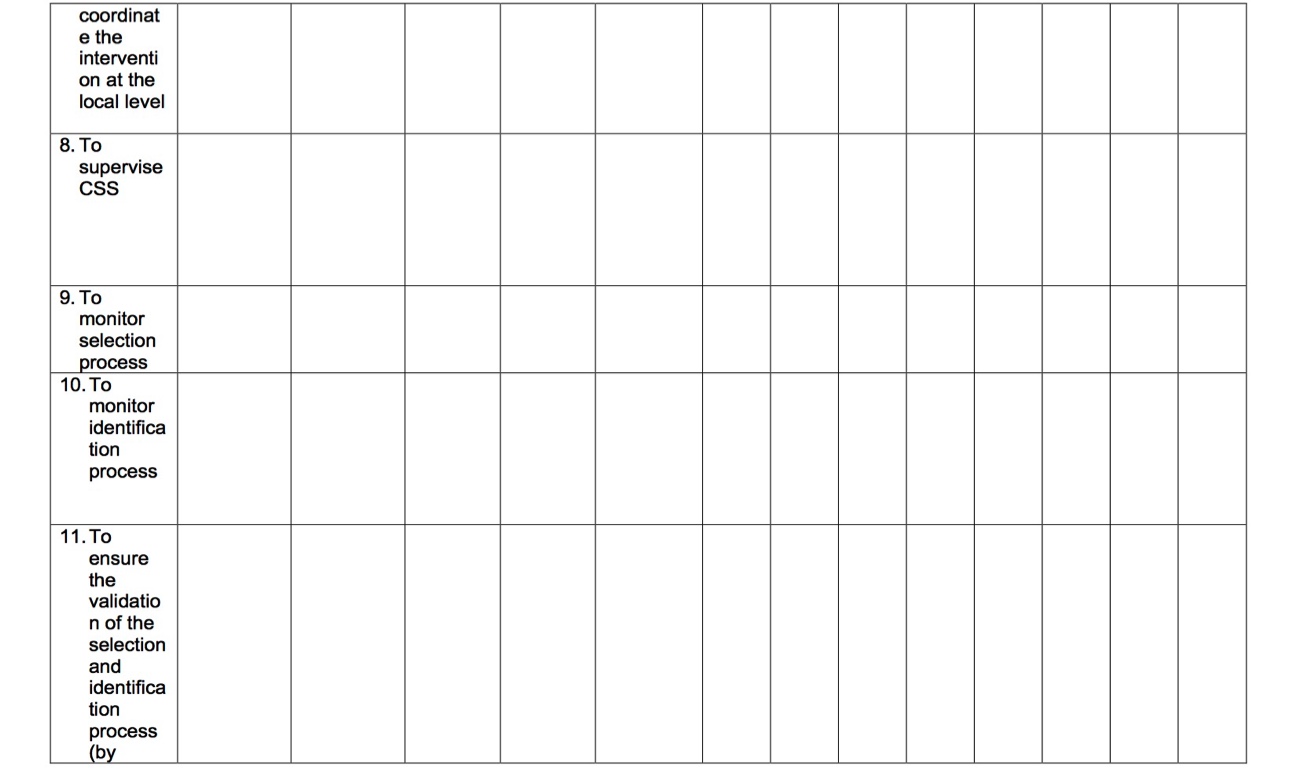


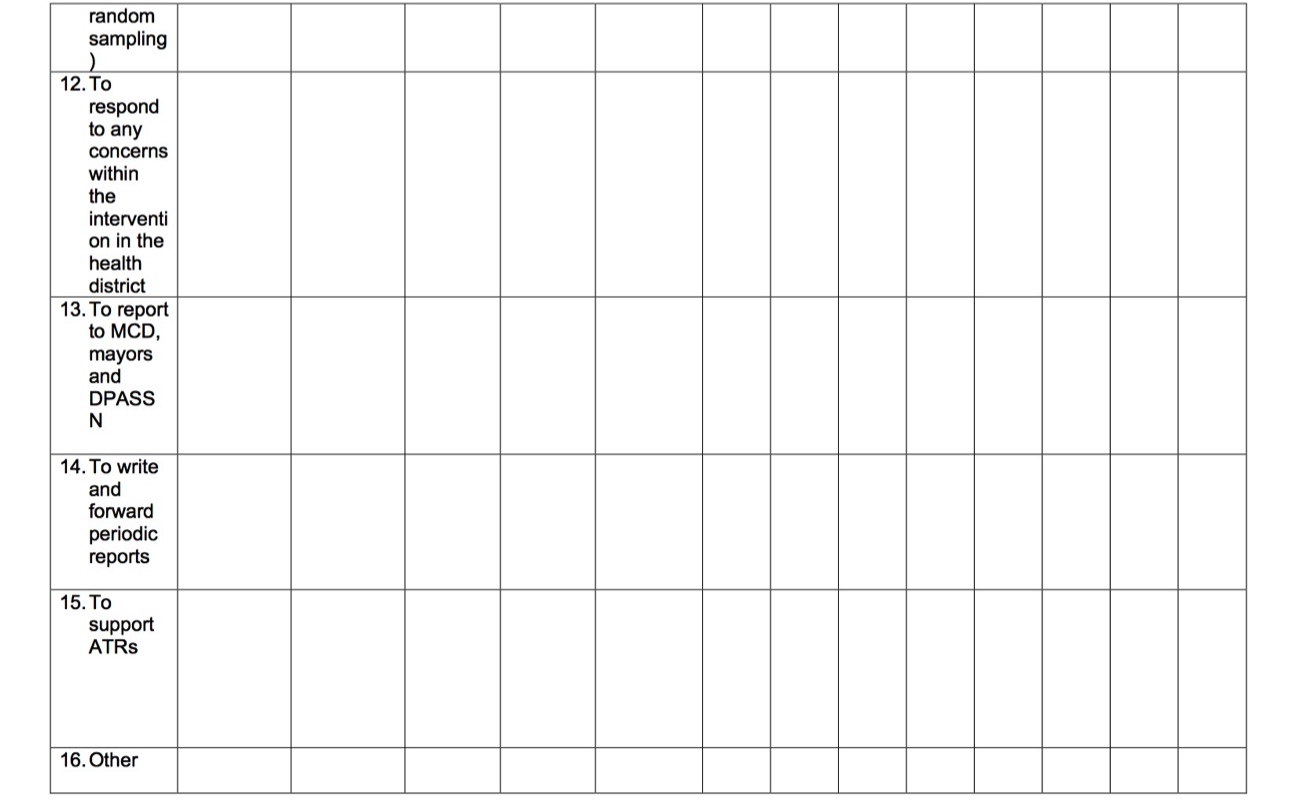


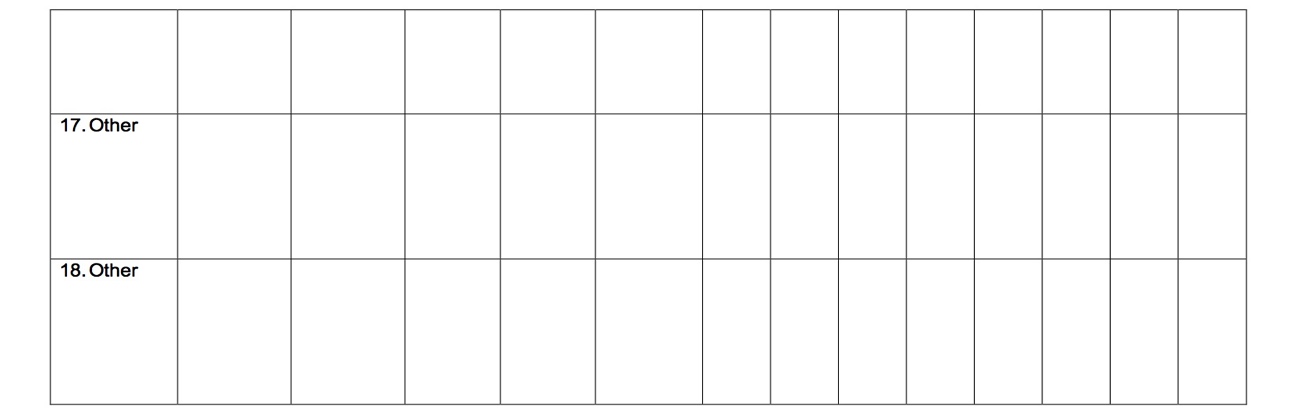

Supplement: Supplementary file 3 — Data Collection Form Regional Technical Assistants (ATR’s). (DOCX 2353 kb) [file 13561_2018_205_MOESM3_ESM.docx]

## **Additional file 4: Self-administered Questionnaire WB**

**
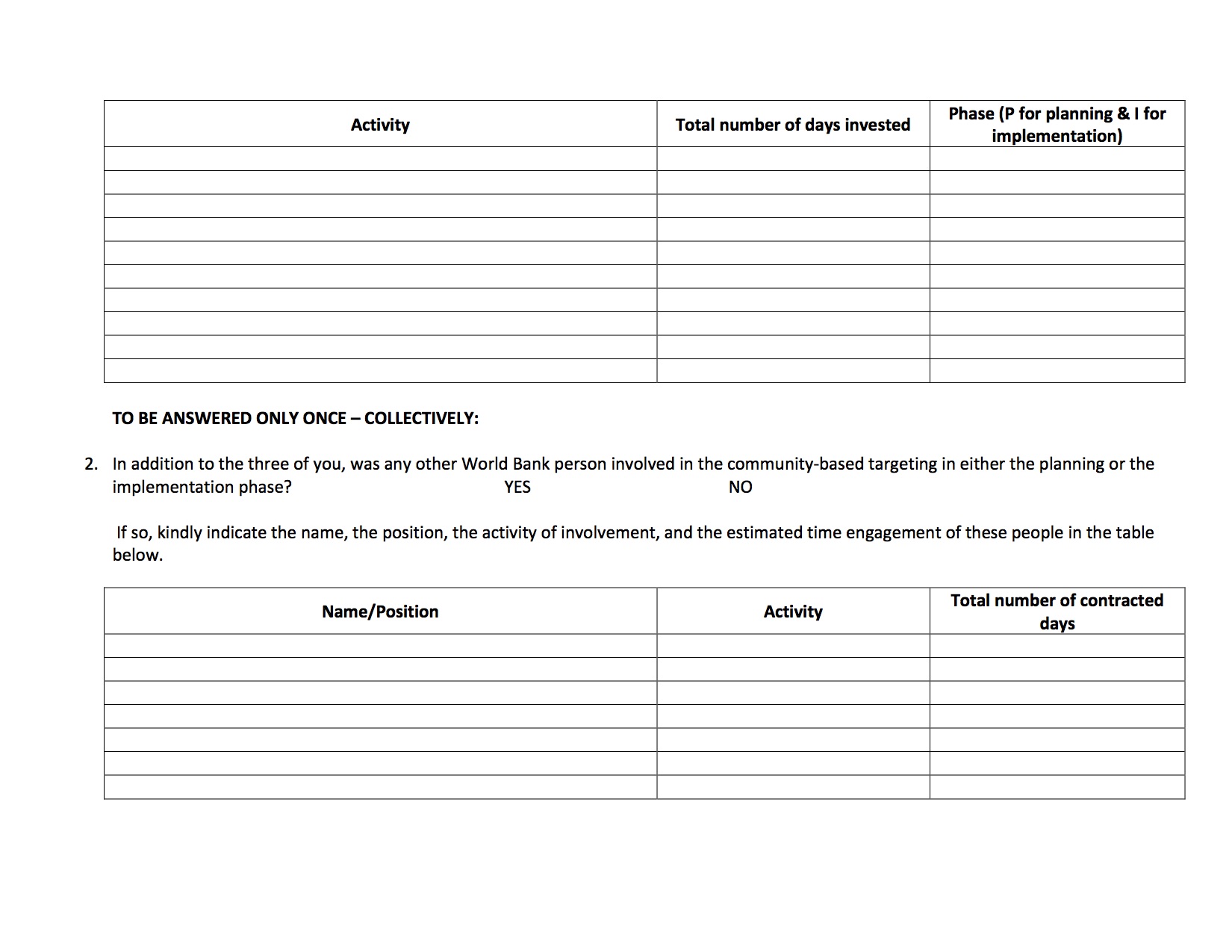
**

Supplement: Supplementary file 4 — Self-administered Questionnaire WB. (DOCX 288 kb) [file 13561_2018_205_MOESM4_ESM.docx]

**Additional file 5: Interview Guide**


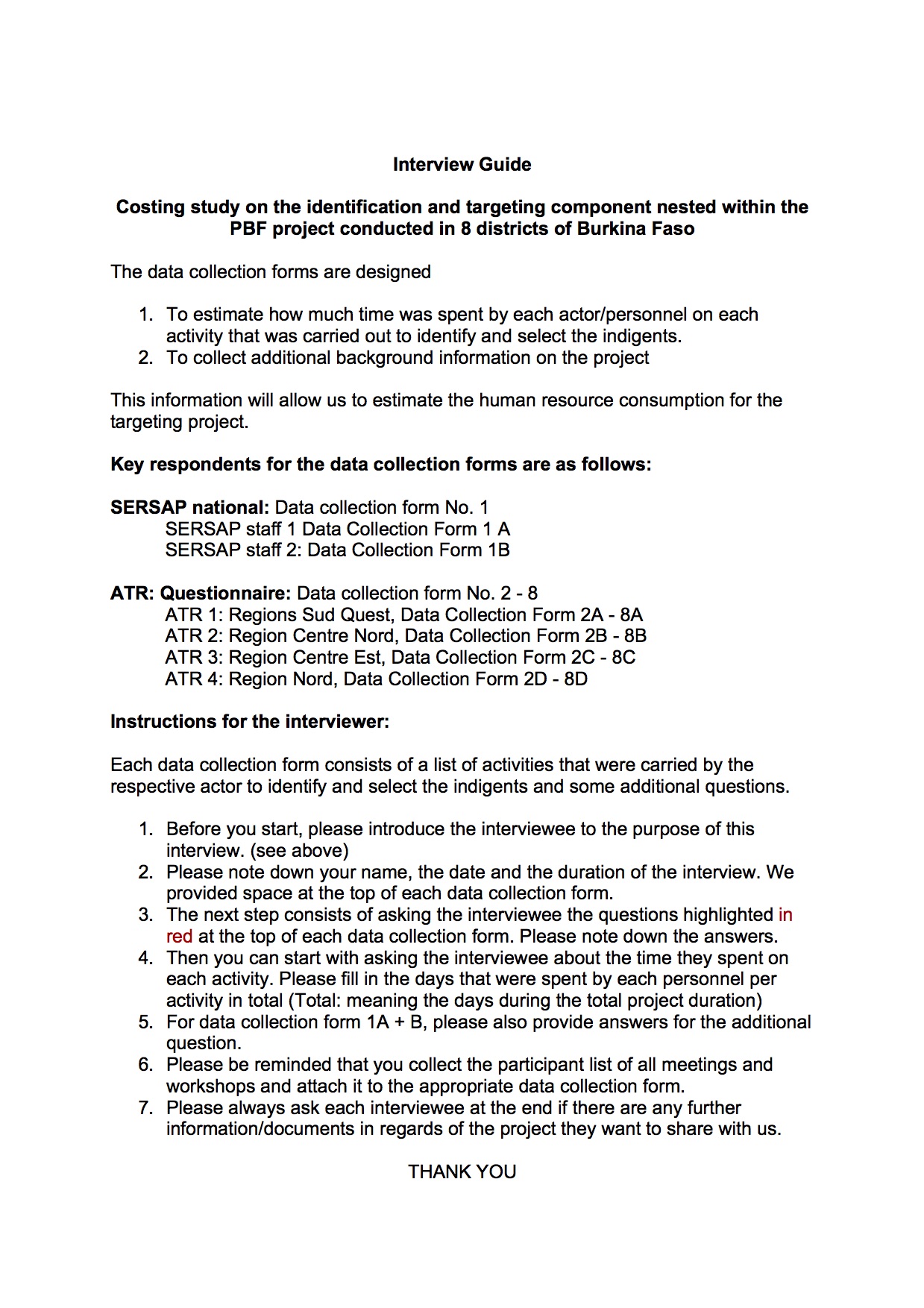

Supplement: Supplementary file 5 — Interview Guide. (DOCX 519 kb) [file 13561_2018_205_MOESM5_ESM.docx]
