# Supplementary material for: How much does community-based targeting of the ultra-poor in the health sector cost? Novel evidence from Burkina Faso
Source: Health Econ Rev. 2018 Sep 4;8:19. doi: 10.1186/s13561-018-0205-7 (PMC6123332; doi:10.1186/s13561-018-0205-7)
Supplement: Supplementary file 6 — Financial and economic costs in USD differentiated between fixed and variable costs and organizational level. (DOCX 24 kb) [file 13561_2018_205_MOESM6_ESM.docx]

**Additional file 6: Financial and economic costs in USD differentiated between fixed and variable costs and organizational level**

| **Fixed Costs** | **Activities** | **Financial Costs inUSD** | **Economic Costs inUSD** |
| --- | --- | --- | --- |
| Central level | 1. General Workshops to define targeting strategy/ develop concept note | 0 | 12,180 |
|  | 2. Initial workshop on targeting the indigents in the PBF project | 0 | 4,921 |
|  | 3. General Coordination/management/Supervision for design phase | 7,098 | 45,306 |
|  | 4. Internal workshop SERSAP | 360 | 625 |
|  | 5. General Coordination/management/Supervision for implementation phase | 10,126 | 41,112 |
|  | 6. Informational meetings at central level | 266 | 4,660 |
|  | 9. National Project launch | 0 | 1,522 |
|  | 15. Designing data collection program, questionnaires, preparing tablets | 24,000 | 24,000 |
|  | 16. Training of data collectors | 10,800 | 10,800 |
|  | 18. Management of the indigent database | 55,535 | 55,535 |
|  | 19. Monitoring & Quality Control | 12,813 | 12,813 |
| **Total Fixed Costs** |  | **120,998** | **213,474** |
| **Variable Costs** | **Activities** |  |  |
| Regional Level | 7. Informational meetings at regional level | 4,410 | 6,187 |
| District Level | 8. Informational meetings at district level | 11,583 | 12,731 |
|  | 10. Training of ELMEO | 17,729 | 18,529 |
| CSPS Level | 11. Training of CSS members | 22,720 | 24,917 |
| Village Level | 14. Selection of the indigents | 96,233 | 403,132 |
|  | 17. Data Collection + Photo taking | 138,541 | 279,550 |
|  | 12. Trainings of GVL member | 757 | 34,673 |
|  | 13. Trainings of CSI member | 46,140 | 82,753 |
| Indigent Level | 20. Production of indigent cards | 88,800 | 88,800 |
|  | 21. Transmission/Distribution of Cards | 39,600 | 48,701 |
| **Total Variable Costs** |  | **466,513** | **999,973** |
| **Grant Total** |  | **587,511** | **1,213,447** |

The differentiation between fixed and variable costs is based on the adoption of the national perspective. However, it is important to consider that the costs of the targeting program vary at different implementation levels (national, regional, district, CSPS, village and indigent-level). Hence, what is variable when considering the national perspective (such as regional costs) is fixed when considering the local perspective. In terms of a possible expansion of the program, we can´t be certain about the extent these costs would remain the same. (i.e. at which level a new scale is reached and hence more resources needed).

We applied the average cost method to calculate the financial and economic cost per region, district, CSPS, village and indigent identified. Thus, the total costs at regional level (FC: USD 4,410; EC: USD 6,187) were divided by the total number of regions included in the targeting program =4; the total costs at district level (FC: 29,312; EC: USD 31,260) were divided by the total number of districts =8; the total costs at CSPS level (FC: USD 22,720; EC: USD 24,917) were divided by the total number of CSPS =198; the total costs at village level (FC: USD 281,671; EC: USD 800,108 ) were divided by the total number of villages =1,172; and the total costs at indigent level (FC: USD 122,353 EC: USD 137,501) were divided by the total number of indigents identified =102,609. This calculation was done first for the financial and then for the economic costs.

**Financial cost function:**

*Cost function using financial* cost

 $C\left( x \right)= F\left( central level \right)+ V\left( region \right)+ V\left( district \right)+V\left( CSPS \right)+ V\left( village \right)+ V\left( indigent \right)$

Where F is Fixed financial costs at the central level

Where V(region) is variable financial costs at the regional level

Where V(district) is variable financial cost at the district level

Where V(CSPS) is variable financial costs at the CSPS level

Where V(Village) is variable financial costs at the village level

Where V(indigent) is variable financial costs at the indigent level

**Economic cost function:**

*Cost function using economic* cost (average)

 $C\left( x \right)= F\left( central level \right)+ V\left( region \right)+ V\left( district \right)+V\left( CSPS \right)+ V\left( village \right)+ V\left( indigent \right)$

Where F is Fixed economic costs at the central level

Where V(region) is variable economic costs at the regional level

Where V(district) is variable economic cost at the district level

Where V(CSPS) is variable economic costs at the CSPS level

Where V(Village) is variable economic costs at the village level

Where V(indigent) is variable economic costs at the indigent level

**Projection of the total financial and economic costs of the targeting program for the entire country:**

Total No of regions in the country: 13

Total No of health districts in the country: 63

Total No of CSPS in the country: 1,495

Total No of villages in the country: 8,438

Total No of indigents in the country? (6 %* of 18.1 million) = 1,086,000

*Based on calculated average of the targeting program

1. *Estimated total financial* costs for expanding the targeting program to the entire nation:

 $C\left( x \right)=120,998 +\left( 1,102.5*13 regions \right)+\left( 3,663,63*63 districts \right)+\left( 114.75*1,495 CSPS \right)+ \left( 240.33*8,438 villages \right)+ (1.25 * 1,086,000 indigents)$

 $C\left( x \right)=120,998 +$14,332.5 + 230,808,69 + 171,551.25 + 2,027,904.54 + 1,357,500

 $C\left( x \right)=$ 3,923,094,98

1. *Estimated total economic* costs for expanding the targeting program to the entire nation:

 $C\left( x \right)=213,474 +\left( 1,546.75*13 regions \right)+\left( 3907,5*63 districts \right)+\left( 125.84 *1495 CSPS \right)+ \left( 682.69*8438 villages \right)+ (1,34 * 1.086.000 indigents)$

 $\boldsymbol{C}\left( \boldsymbol{x} \right)\mathbf{=}$213,474 + 20,107.75 + 246,172.5 + 188,130.8 + 5,760,538.22 + 1,455,240

 $\mathbf{C}\left( \mathbf{x} \right)\mathbf{=}$ 7,883,663.27
