# Supplementary material for: How much does community-based targeting of the ultra-poor in the health sector cost? Novel evidence from Burkina Faso
Source: Health Econ Rev. 2018 Sep 4;8:19. doi: 10.1186/s13561-018-0205-7 (PMC6123332; doi:10.1186/s13561-018-0205-7)
Supplement: Supplementary file 7 — Sensitivity Analysis: Wage Informal Workers (in USD). (DOCX 14 kb) [file 13561_2018_205_MOESM7_ESM.docx]

**Additional file 7: Sensitivity Analysis: Wage Informal Workers (in USD)**

| **Activity** | **Economic Costs**  (Application of Minimum Wage) in USD | **Economic Costs**  (Application of Average Wage) in USD |
| --- | --- | --- |
| *Design Phase* |  |  |
| General Coordination/Management | 62,174 | 62,174 |
| *Implementation Phase* |  |  |
| General Coordination/Management | 63,177 | 63,189 |
| Training | 159,824 | 164,102 |
| Selection of the ultra-poor | 392,060 | 423,064 |
| Data Collection | 328,958 | 343,608 |
| Card Production/ Distribution | 116,101 | 116,663 |
| M&E | 11,339 | 11,339 |
| Overhead | 79,814 | 79,814 |
| **Total** | **1,213,447** | **1,263,953** |
