# Supplementary material for: How much does community-based targeting of the ultra-poor in the health sector cost? Novel evidence from Burkina Faso
Source: Health Econ Rev. 2018 Sep 4;8:19. doi: 10.1186/s13561-018-0205-7 (PMC6123332; doi:10.1186/s13561-018-0205-7)
Supplement: Supplementary file 8 — Sensitivity Analysis: Increase and Decrease of overhead costs (in USD). (DOCX 16 kb) [file 13561_2018_205_MOESM8_ESM.docx]

**Additional file 8: Sensitivity Analysis: Increase and Decrease of overhead costs (in USD)**

| **Activity** | **Economic Costs** (Overhead: SERSAP 13%, WB 20%) **Baseline** | **Economic Costs**  (Overhead:  SERSAP 16%; WB 22 %) | **Economic Costs**  (Overhead: SERSAP 18%; WB 24%) | **Economic Costs**  (Overhead:  SERSAP 10%; WB 18 %) | **Economic Costs**  (Overhead: SERSAP 7%; WB 16%) |
| --- | --- | --- | --- | --- | --- |
| *Design Phase* |  |  |  |  |  |
| General Coordination/Management | 62,174 | 62,174 | 62,174 | 62,174 | 62,174 |
| *Implementation Phase* |  |  |  |  |  |
| General Coordination/Management | 63,177 | 63,177 | 63,177 | 63,177 | 63,177 |
| Training | 159,824 | 159,824 | 159,824 | 159,824 | 159,824 |
| Selection of the ultra-poor | 392,060 | 392,060 | 392,060 | 392,060 | 392,060 |
| Data Collection | 328,958 | 328,958 | 328,958 | 328,958 | 328,958 |
| Card Production/ Distribution | 116,101 | 116,101 | 116,101 | 116,101 | 116,101 |
| M&E | 11,339 | 11,339 | 11,339 | 11,339 | 11,339 |
| Overhead | 79,814 | 93,072 | 106,329 | 66,557 | 53,299 |
| SERSAP | 40,346 | 49,657 | 58,968 | 31,036 | 21,725 |
| World Bank | 39,468 | 43,415 | 47,361 | 35,521 | 31,574 |
| **Total** | **1,213,447** | **1,226,705** | **1,239,962** | **1,200,190** | **1,186,932** |
