# Supplementary material for: How much does community-based targeting of the ultra-poor in the health sector cost? Novel evidence from Burkina Faso
Source: Health Econ Rev. 2018 Sep 4;8:19. doi: 10.1186/s13561-018-0205-7 (PMC6123332; doi:10.1186/s13561-018-0205-7)
Supplement: Supplementary file 9 — Sensitivity Analysis: Increase and decrease SERSAP Budget (in USD). (DOCX 15 kb) [file 13561_2018_205_MOESM9_ESM.docx]

**Additional file 9: Sensitivity Analysis: Increase and decrease SERSAP Budget (in USD)**

| **Activity** | **Economic Costs (Baseline)** | **Increased by 5 %** | **Increased by 10 %** | **Increased by 15 %** | **Increased by 20 %** | **Decreased by 5 %** | **Decreased by 10 %** | **Decreased by 15 %** | **Decreased by 20 %** |
| --- | --- | --- | --- | --- | --- | --- | --- | --- | --- |
| *Design Phase* |  |  |  |  |  |  |  |  |  |
| General Coordination/Management | 62,174 | 62,505 | 62,834 | 63,164 | 63,494 | 61,845 | 61,515 | 61,185 | 60,855 |
| Implementation Phase |  |  |  |  |  |  |  |  |  |
| General Coordination/Management | 63,177 | 64,344 | 65,512 | 66,679 | 67,847 | 62,009 | 60,842 | 59,674. | 58,507 |
| Training | 159,824 | 163,689 | 167,554 | 171,418 | 175,283 | 155,959 | 152,094. | 148,229 | 144,364 |
| Selection of the ultra-poor | 392,060 | 396,318 | 400,576 | 404,834 | 409,092 | 387,801 | 383,543 | 379,285 | 375,027 |
| Data Collection | 328,958 | 334,288 | 339,618 | 344,949 | 350,279 | 323,627 | 318,297 | 312,966 | 307,636 |
| Card Production & Distribution | 116,101 | 116,101 | 116,101 | 116,101 | 116,101 | 116,101 | 116,101 | 116,101 | 116,101 |
| M&E | 11,339 | 11,906 | 12,473 | 13,040 | 13,607 | 10,772 | 10,205 | 9,638 | 9,071 |
| Overhead | 79,814 | 81,832 | 83,849 | 85,866 | 87,847 | 77,797 | 75,780 | 73,762 | 71,745 |
| **Total** | **1,213,447** | **1,230,983** | **1,248,517** | **1,266,051** | **$1,283,550** | **1,195,911** | **1,178,377** | **$1,160,840** | **1,143,306** |
